# Subgrouping breast cancer patients based on immune evasion mechanisms unravels a high involvement of transforming growth factor-beta and decoy receptor 3

**DOI:** 10.1371/journal.pone.0207799

**Published:** 2018-12-04

**Authors:** Mayassa J. Bou-Dargham, Yuhang Liu, Qing-Xiang Amy Sang, Jinfeng Zhang

**Affiliations:** 1 Department of Chemistry and Biochemistry, Florida State University, Tallahassee, Florida, United States of America; 2 Department of Statistics, Florida State University, Tallahassee, Florida, United States of America; 3 Institute of Molecular Biophysics, Florida State University, Tallahassee, Florida, United States of America; University of Tennessee Health Science Center, UNITED STATES

## Abstract

In the era of immunotherapy and personalized medicine, there is an urgent need for advancing the knowledge of immune evasion in different cancer types and identifying reliable biomarkers that guide both therapy selection and patient inclusion in clinical trials. Given the differential immune responses and evasion mechanisms in breast cancer, we expect to identify different breast cancer groups based on their expression of immune-related genes. For that, we used the sequential biclustering method on The Cancer Genome Atlas RNA-seq breast cancer data and identified 7 clusters. We found that 77.4% of the clustered tumor specimens evade through transforming growth factor-beta (TGF-β) immunosuppression, 57.7% through decoy receptor 3 (DcR3) counterattack, 48.0% through cytotoxic T-lymphocyte-associated protein 4 (CTLA4), and 34.3% through programmed cell death-1 (PD-1). TGF-β and DcR3 are potential novel drug targets for breast cancer immunotherapy. Targeting TGF-β and DcR3 may provide a powerful approach for treating breast cancer because 57.7% of patients overexpressed these two molecules. Furthermore, triple-negative breast cancer (TNBC) patients clustered equally into two subgroups: one with impaired antigen presentation and another with high leukocyte recruitment but four different evasion mechanisms. Thus, different TNBC patients may be treated with different immunotherapy approaches. We identified biomarkers to cluster patients into subgroups based on immune evasion mechanisms and guide the choice of immunotherapy. These findings provide a better understanding of patients’ response to immunotherapies and shed light on the rational design of novel combination therapies.

## Introduction

Cancer cells express markers that differentiate them from normal cells and allow for their detection through immune surveillance and subsequent destruction [1–3]. Antigen-presenting cells (APCs) phagocytose and present tumor antigens on their cell surface together with class I major histocompatibility complex (MHC-I): HLA-A and HLA-B. The recognition of antigen-MHC-I complexes by naïve cytotoxic T lymphocytes (CTLs; *aka* CD8^+^ T cells) results in their activation. Then, trafficking and infiltration of CTLs from the bloodstream to the tumor microenvironment follow, orchestrated by adhesion molecules. Activated CTLs kill cancer cells via (i) granular exocytosis: perforins and granzymes or (ii) death ligand-death receptor-mediated apoptosis. The latter occurs through the binding of death ligand cluster of differentiation 95 (CD95L; *aka* FasL) to the death receptor CD95 (Fas) on tumor cell surface [4,5]. This process from antigen presentation to apoptosis induction by CTL is known as the cancer-immunity cycle [6].

Despite immune surveillance, cancer cells manage to evade immune destruction [7]. There are multitudes of evasion mechanisms that govern the suppressive nature of the tumor microenvironment and understanding these mechanisms is an active area of study. Several clinical trials targeting the molecules that inhibit anti-tumor immune response in breast cancer are in progress (S1 File). Given the heterogeneity of breast cancer, several studies have been conducted to investigate the molecular and prognostic differences between the different hormone receptor subtypes and the ductal and lobular subgroups. Triple-negative breast cancer (TNBC) studies have identified intra-subtype heterogeneity where certain patients showed poor prognosis while others responded well to anthracycline-based treatments [8]. Scientific evidence suggested that the clinical outcome for TNBC is affected by tumor-infiltrating immune cells [8,9]. Studies on invasive breast cancer showed that invasive lobular carcinoma (ILC) is 3 times more likely to metastasize and less responsive to neoadjuvant chemotherapy compared to invasive ductal carcinoma (IDC) [10,11]. Furthermore, an “immune-related” subgroup of ILC was identified, characterized by upregulated mRNA expression of PD-1, it’s ligand PD-L1, and CTLA4 [12].

Currently, immune checkpoints, CTLA4 and PD-1/PD-L1, are the most intensively studied immune evasion molecules in cancer, and immunotherapies targeting them are probably the most successful ones. However, substantial proportions of PD-L1 positive or CTLA4 positive patients do not respond to the corresponding immunotherapies [13,14]. Thus, these molecules are not reliable biomarkers for the prediction of treatment response [15].

Furthermore, clinical trials are shifting towards combination immunotherapy under the assumption that multiple immune evasion mechanisms may be utilized by a tumor. However, the choice of immunotherapy and combined treatments is poorly guided as they are generally given indiscriminately even though different patients may have different evasion mechanisms.

To address the current knowledge gaps, we sought to gain further insight into immune evasion mechanisms and heterogeneity in breast cancer and provide a better diagnostic to help guide treatments. We used the sequential biclustering method and classification tree algorithm to analyze the expression pattern of The Cancer Genome Atlas (TCGA) RNA-seq data of immune-related genes and identify putative biomarkers, respectively. We identified seven distinct breast cancer groups that represent different combinations of evasion mechanisms (M) and reveal molecular features that provide a better understanding of the evasion mechanisms in breast cancer and suggest potential therapeutic strategies.

## Materials and methods

### Generating the list of immune genes

**Fig 1:**
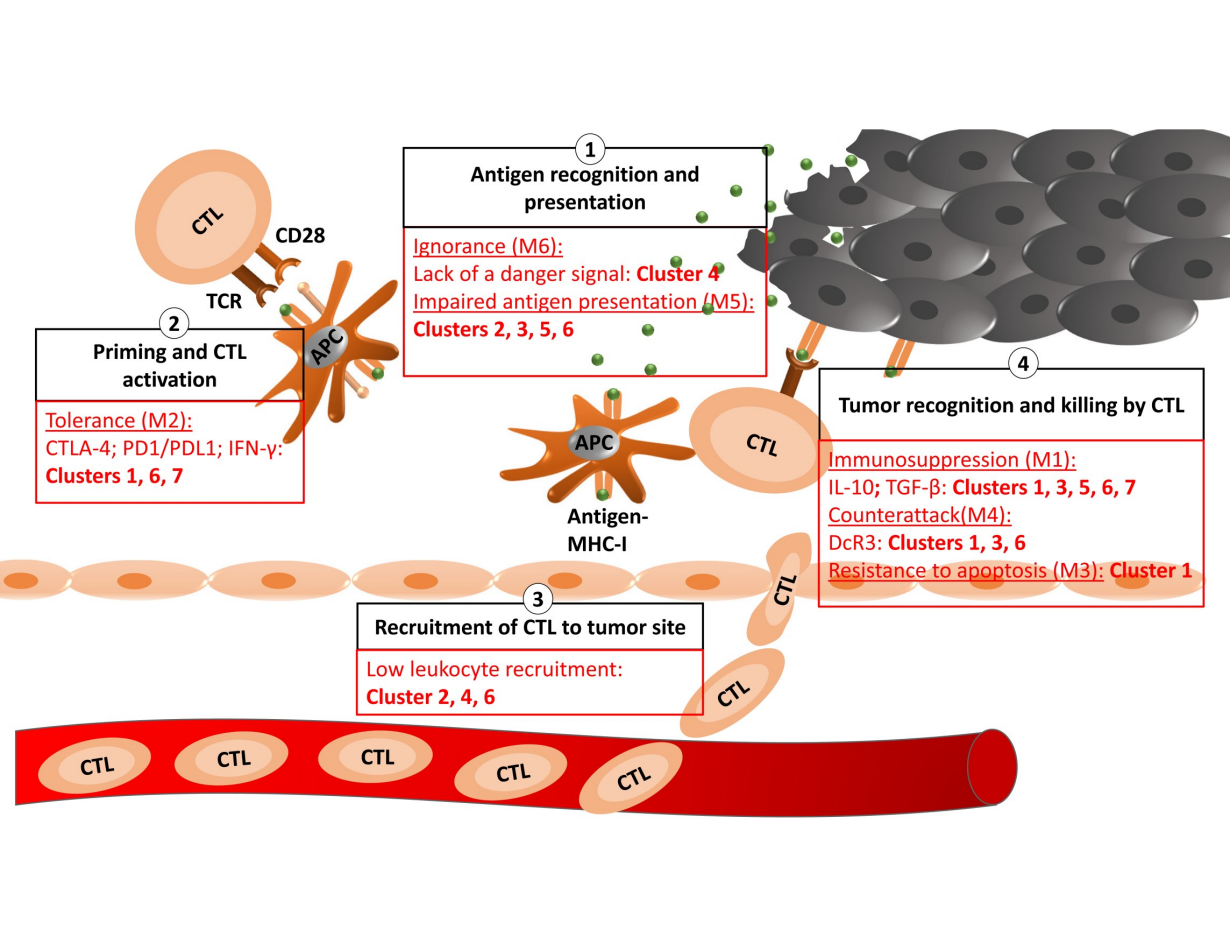
Evasion at different levels of the cancer-immunity cycle in each cluster.

Based on the current knowledge of the mechanism of tumor evasion from immune system destruction, a list of 86 genes was generated manually based on the available literature (S2 File). The generated list included genes involved in the cancer-immunity cycle, and tolerance and immunosuppression-inducing genes (Fig 1). To make sure no important genes were missing, the list was expanded from 86 genes to 1,356 by adding all interacting proteins determined using bioGRID database (https://thebiogrid.org/) [16].

### TCGA breast cancer dataset

RNA-seq data of 1,065 breast cancer (BRCA) tumor samples were obtained from TCGA (http://cancergenome.nih.gov/) together with 111 non-malignant adjacent normal tissue samples. So, we created two data matrices: cancer matrix (1356*1065) and normal matrix (1356*111). The patients’ clinical information was obtained as well from TCGA and matched with their genomic information.

### Sequential biclustering

**Fig 2:**
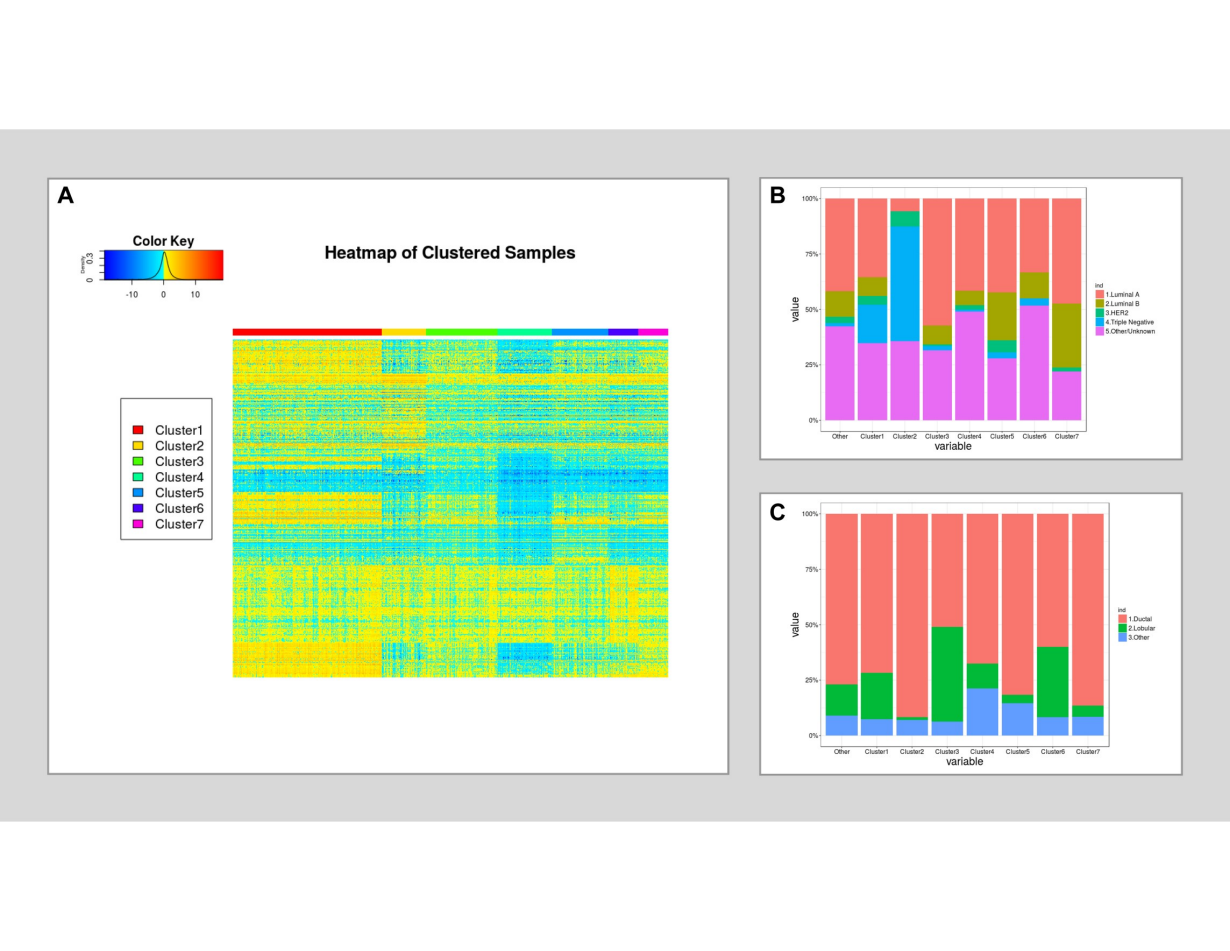
The clustering results for the sequential biclustering. (A) The heatmap representing the level of gene expression (rows) in different clusters of patients (columns). (B) The percentage of different breast cancer receptor subtype and (C) lobular and ductal carcinomas, ILC and IDC, per cluster.

Biclustering, also known as block clustering, co-clustering, or two-way clustering, is the technique of simultaneously clustering the rows and columns of a matrix. In our study, biclustering shuffles rows (genes) and columns (patients) of the data matrix to generate clusters with a minimum variation of gene expression amongst a group of patients (intra-cluster variation) and maximum variation with other groups of patients (inter-cluster variation). We used the *biclust* package available in R on the Log2-transformed gene expression data of cancer patients to divide patients into subgroups based on their expression of immune-related genes. The BCPlaid algorithm was used as it can cluster patients (columns) based on their similarity in gene expression (row-based) rather than by the similar gene expression per patient (column-based) [17] (Fig 2A).

Running the BCPlaid algorithm generated overlapping clusters of patients and genes, where the clusters are sorted by layers output by the program. Each layer is searched based on residuals given all the previous layers, so by nature, earlier layers contain more information about the data and tend to be more coherent. Since our goal is to divide patients into non-overlapping groups, which means that one patient should fall in only one group, we adopted a sequential approach. In this procedure, we run the BCPlaid algorithm multiple times sequentially. After each run, we take the earliest cluster with at least 5% of the cancer patients and remove the patients in this cluster from the whole dataset. The remaining dataset will be clustered in the next run. The constraint of each candidate cluster having at least 5% of the cancer patients guarantees that finally, each immune evasion cluster is a representative set of patients from the population.

### Verification of robustness of the biclustering procedure

The robustness (reproducibility) of the proposed sequential biclustering procedure was assessed by running the whole procedure multiple times with five different random seeds. We reran the procedure with each of these seeds and checked the agreement, measured by the random index, between the clusters obtained by these runs and the original ones [18]. Specifically, each seed leads to a set of patient group labels *C*(*i*),*i* = 1,2,…,*n*, where *n* is the total number of patients. Denote the original group labels as *C*_0_(*i*) and the group labels from the other five runs as *C*_*k*_(*i*),*k* = 1,2,3,4,5. For each pair of patients 1 ≤ *i* < *j* ≤ *n*, define

$$\delta_{k}^{ij}=\{\begin{array}{l} 1,ifC_{0}\left(i\right)=C_{0}\left(j\right),C_{k}\left(i\right)=C_{k}\left(j\right)\vee C_{0}\left(i\right)\neq C_{0}\left(j\right),C_{k}\left(i\right)\neq C_{k}\left(j\right)\\ \;0,otherwise \end{array}$$

*and* $\gamma_{k}=\frac{\sum_{1\leq i<j\leq n}\delta_{k}^{ij}}{\left(\begin{array}{c} n\\ 2 \end{array}\right)},k=1,2,3,4,5$. This is an intuitive measure of consistency between two different sequences of biclustering runs by investigating if any two patients are clustered consistently. Values of agreement with the five random number seeds were all in the range of 90–93% (S3 File). We also compared the results of each seed with the classification tree mentioned below, and error rates were relatively stable too (S3 File).

### Fisher exact test

In our study, we wanted to determine whether the clustering was dependent on breast cancer receptor status and invasive ductal and lobular subtypes. Accordingly, the receptor status information was obtained from TCGA and a Fisher exact test was used (Tables 1 and 2). The p-values were calculated by comparing the number of patients in a cluster belonging to a specific subtype to the total number of patients in the cluster. A p-value ≤ 0.05 indicates that distribution of a number of patients in that cluster is significantly different from the overall pattern.

**Table 1:** The association of clusters with breast cancer subtypes (receptor status).

Cluster	Number of patients	HER2	Luminal A	Luminal B	TNBC	Fisher exact test p-value
**Cluster 1**	193	12 (6.2%)	105 (54.4%)	25 (13.0%)	51 (26.4%)	8.46E-03
**Cluster 2**	56	6 (10.7%)	5 (8.9%)	0	45 (80.4%)	4.95E-26
**Cluster 3**	98	1 (1%)	82 (83.7%)	12 (12.2%)	3 (3%)	3.10E-05
**Cluster 4**	55	2 (3.6%)	45 (81.8%)	7 (12.7%)	1 (1.8%)	4.49E-03
**Cluster 5**	80	6 (7.5%)	47 (58.75%)	24 (30%)	3 (3.75%)	9.30E-04
**Cluster 6**	29	0	20 (68.9%)	7 (24%)	2 (6.9%)	0.315
**Cluster 7**	46	1 (2%)	28 (60.9%)	17 (37%)	0	1.94E-04
**Other**	201	6 (3.0%)	84 (41.8%)	23 (11.4%)	3 (1.5%)	2.16E-04

The results of the Fisher exact test show a significant association between cluster 3, cluster 4, cluster 5, cluster 7 and Luminal A, and CL2 and triple negative breast cancer (TNBC) (P<0.05). Some patients had no information on their receptor status in TCGA, hence the lower number of patients in column 2 compared to the identified. HER2: Human epidermal growth factor receptor 2.

**Table 2:** The association of clusters with invasive lobular and ductal carcinoma subgroups, ILC and IDC, respectively.

Cluster	Number of patients	IDC	ILC	Fisher exact test p-value
**Cluster 1**	274	212 (77.37%)	62 (22.63%)	3.5030E-01
**Cluster 2**	79	78 (98.73%)	1 (1.27%)	1.4315E-06
**Cluster 3**	134	73 (54.48%)	61 (45.52%)	6.2561E-10
**Cluster 4**	85	73 (85.88%)	12 (14.12%)	2.5155E-01
**Cluster 5**	93	89 (95.7%)	4 (4.30%)	6.2294E-05
**Cluster 6**	55	36 (65.45%)	19 (34.55%)	1.5219E-02
**Cluster 7**	54	51 (94.44%)	3 (5.56%)	6.8520E-03
**Other**	182	154 (84.62%)	28 (15.38%)	1.8154E-01

Clusters 2, 5 and 7 were significantly associated with IDC and clusters 3 and 6 with ILC.

### Differential gene expression analysis

Comparison of gene expression between any two groups is done by a combination of p-value from t-test and log2 fold change cutoff. Both comparisons for tumor versus tumor and tumor versus normal were performed. T-tests were implemented to compare the mean expression of a gene within a cluster to the mean expression of other cancer patients (tumor versus tumor) and to that of normal samples (tumor versus normal) (S4 File). Comparing tumor to tumor can be misleading as a gene may be up-regulated for example in one tumor cluster compared to others but is still less than normal. Thus, it is important to use the normal as a reference. The number of genes per cluster may not always be enough to help determine the mechanism of evasion. Knowing that each cluster of patients will have different levels of gene expression, we unified all the genes (rows) from the seven clusters and checked for their mean expression per cluster of patients.

### Pathway analysis

**Fig 3:**
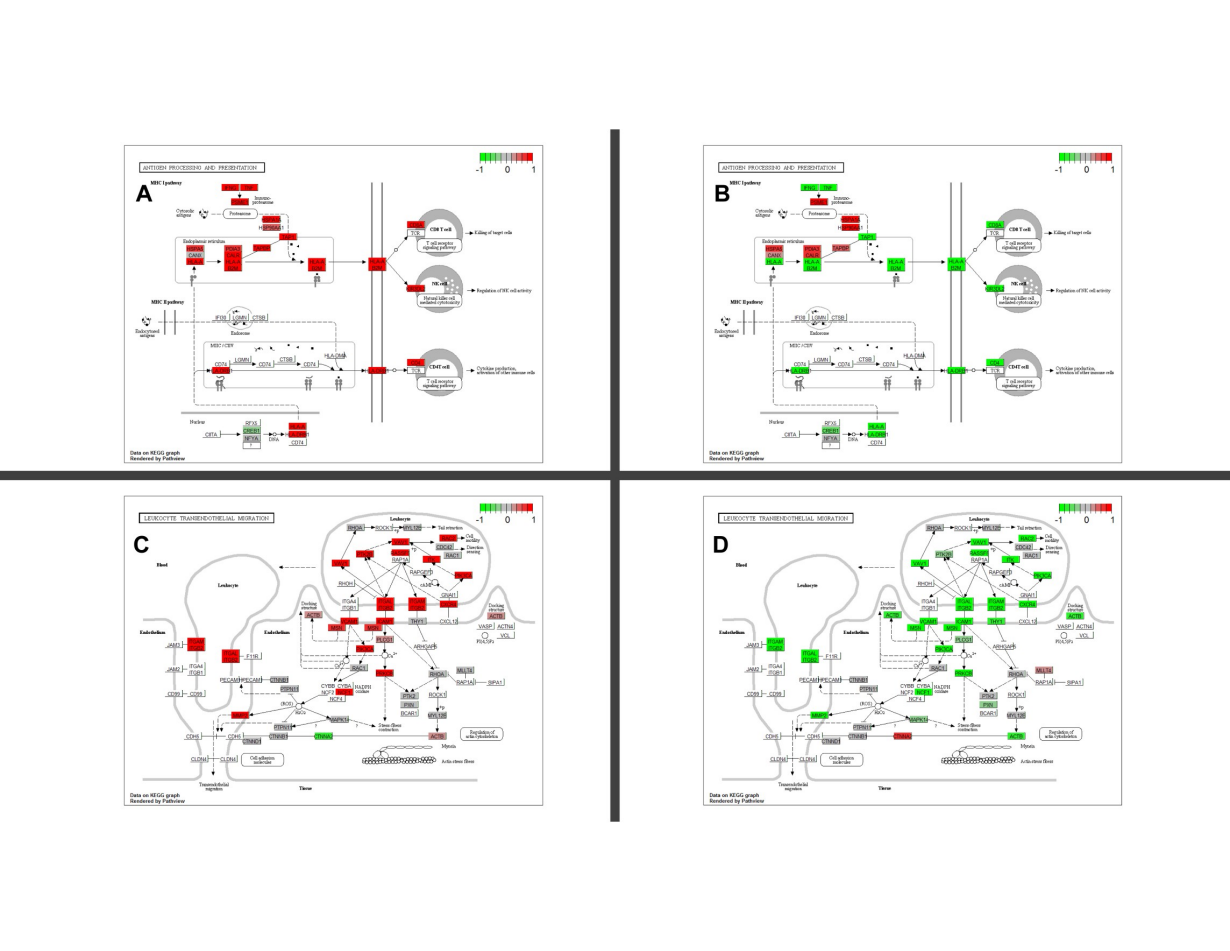
Pathway analysis based on the log2 fold change for clusters 1 and 4 compared to normal. Fold change level of molecules involved in antigen processing and presentation molecules in cluster 1 (A) and cluster 4 (B). Fold change level of molecules involved in leukocyte recruitment in cluster 1 (C) and cluster 4 (D). These results show how cluster 1 genes for the first 2 steps of the cancer-immunity cycle are up-regulated while those of cluster 4 are mostly downregulated. These pathways and others for other clusters and steps of the cancer-immunity pathway are in S5 File. The color scale ranges from the downregulated expression in green (-1 fold), to the non-differential expression in grey, to the up-regulated expression in red (+1 fold).

To find out whether an immune pathway is altered in each of the 7 clusters, we compared the expression of genes in a cluster to those outside that cluster and normal samples (figures names are Tum_Tum and Tum_Nor, respectively). The significantly differentially expressed genes and the corresponding KEGG pathways were plotted using the R/Bioconductor package, *pathview* for visualization [19]. We chose the immune-related pathways and showed only the pathways for antigen processing and presentation (hsa04612), leukocyte transendothelial migration (hsa04670), and cell adhesion molecules (hsa04514) that also shows the fold change of PD-1 (PDCD1), PD-L2 (PDCD1LG2), and CTLA4 (S5 File and Fig 3). KEGG however lacks immune evasion pathways. Thus, we categorized the genes into different groups based on their role in the cancer-immunity cycle (S6 File). Using the p-values generated by the t-test for the tumor-tumor and tumor-normal mean comparisons, we were able to understand at which level evasion was happening and using which molecules.

### Classification tree

**Fig 4:**
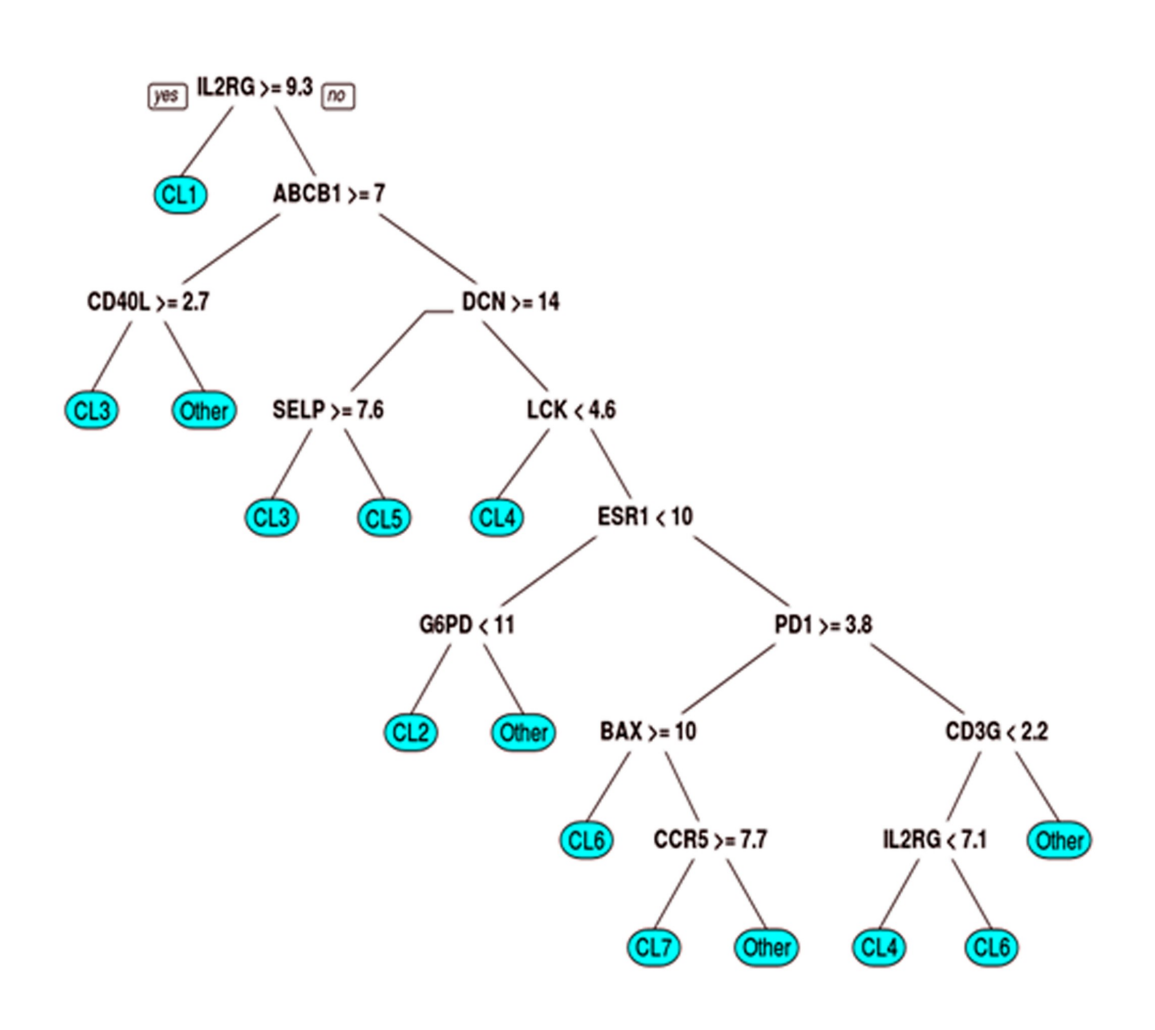
Classification tree with 12 biomarkers and their log2 gene expression cutoffs for the identified clusters (CL). Interleukin-2 receptor subunit gamma (IL2RG), ATP-binding cassette sub-family B member 1 (ABCB1), cluster of differentiation-40 ligand (CD40LG), decorin (DCN), lymphocyte-specific protein tyrosine kinase (LCK), selectin-P (SELP), estrogen receptor-1 (ESR1), glucose-6-phosphate dehydrogenase (G6PD), programmed cell death-1 (PDCD1), cluster of differentiation-3 subunit gamma.

After obtaining the 7 clusters, we wanted to identify a small set of biomarker genes which could classify a subset of patients into their corresponding clusters. A classification tree was used to build a model to predict the cluster into which a patient sample belongs. This was achieved using the *rpart* package in R [20,21]. The selected biomarker genes with their cutoff values are displayed in Fig 4.

## Results

### Cohort and patient clustering

To investigate the different evasion mechanisms in breast cancer, we first compiled a list of 1,356 genes involved in immune activation and immune evasion (S2 File) as described in the Methods section. Then the RNA-seq expression data of these genes in breast cancer patients were obtained from TCGA database and used to categorize patients into different groups using a sequential biclustering algorithm based on BCPlaid [17]. Eighty-one percent of TCGA breast cancer patients were clustered into 7 groups with non-overlapping patients, whereas the other nineteen percent fell into much smaller groups whose specific expression patterns were not characterized in this study (S2 File). TCGA’s 111 nonmalignant adjacent breast cancer samples were used as a normal reference for gene expression.

### Identifying evasion mechanisms in the seven clusters

To understand the mechanisms of evasion of patients falling into a specific group, the mean expression and fold change of genes involved in different steps of cancer-immunity cycle were compared to those of normal samples (S4 and S6 Files). The evasion mechanisms (M) were identified based on the rationale summarized in Table 3 and discussed in S7 File.

**Table 3:** The rationale for deciding the immune evasion mechanisms (M).

Mechanism	Genes	Expression compared to normal
**Immunosuppression (M1)**	IL-10 or TGF-β1 or TGF-β2	Up-regulated
**Tolerance (M2)**	CTLA4 or (PD-1 and PD-L1/2) or IFN-γ	Up-regulated
**Anti-apoptosis (M3)**	At least 2 out of {BIRC3, TNFAIP3, TRAF1, TRAILR4}	Up-regulated
**Counterattack (M4)**	DcR3	Up-regulated
**Impaired antigen presentation (M5)**	B2M or HLA-A or HLA-B	Not up-regulated
	and CD4 or CD8A	Not up-regulated
	and at least 1 out of {GZMA, GZMB, PRF1}	Up-regulated
	or TGF-β1	Up-regulated
**Ignorance (M6)**	B2M and HLA-A and HLA-B	Not up-regulated
	and CD4 and CD8A	Not up-regulated
	and GZMA and GZMB and PRF1	Not up-regulated
	and TGF-β1	Not up-regulated

Interleukin 10 (IL-10), transforming growth factor-beta (TGF-β1/2), cytotoxic T-lymphocyte associated protein 4 (CTLA4); programmed cell death-1 (PD-1) and ligand (PD-L1/2), interferon gamma (IFN-γ), Baculoviral IAP repeat-containing protein 3 (BIRC3), Tumor necrosis factor alpha-induced protein 3 (TNFAIP3), TNF receptor-associated factor 1 (TRAF1), TNF-related apoptosis-inducing ligand receptor 4 (TRAILR4), decoy receptor 3 (DcR3); beta 2 microglobulin (B2M), human leukocyte antigen A and B (HLA-A/B), cluster of differentiation 4 (CD4), cluster of differentiation 8A (CD8A), granzyme A and B (GZMA/B), and perforin 1 (PRF1).

### Evident immunosuppression with TGF-β1 and TGF-β1 + DcR3

Although several clusters had different combinations of evasion mechanisms, the majority of breast cancer patient groups shared and upregulated expression TGF-β1. Around 77.4% of the clustered TCGA breast cancer patients’ evasion was through TGF-β1-mediated immunosuppression, 57.7% with DcR3, 48.0% with CTLA4, and 34.3% with PD-1. All clusters with upregulated DcR3 (57.7%) had upregulated TGF-β1 expression as well and all 34.3% with upregulated PD-1 had upregulated CTLA4 expression (Table 4). Thus, the most prevalent evasion mechanisms are through TGF-β1 and TGF-β1 and DcR3 combined.

**Table 4:** The mechanism of evasion in each cluster and the potential immunotherapies for future clinical trials.

Cluster	Mechanism of Evasion	Potential Immunotherapies
**Cluster 1 (TNBC)**	M1: Immunosuppression: IL-10, TGF-β1	Anti-TGF-β1
	M2: Tolerance: CTLA4, PD-1/PD-L1, IFN-γ	Anti-PD-1; anti-CTLA4; anti-IFN-γ*
	M3: Apoptosis resistance: Anti-apoptotic molecules^¥^	-
	M4: Counterattack: DcR3	Anti-DcR3
**Cluster 2 (TNBC, IDC)**	M5: Impaired antigen presentation	DC vaccine + Chemotherapy
**Cluster 3 (Luminal A, ILC)**	M1: Immunosuppression: TGF-β1	Anti-TGF-β1
	M4: Counterattack: DcR3	Anti-DcR3
	M5: Impaired antigen presentation	DC vaccine
**Cluster 4 (Luminal A)**	M6: Ignorance: No danger signals	DC vaccine + Chemotherapy
**Cluster 5 (Luminal B, IDC)**	M1: Immunosuppression: TGF-β1	Anti-TGF-β1
	M5: Impaired antigen presentation	DC vaccine
**Cluster 6 (ILC)**	M1: Immunosuppression: TGF-β1	Anti-TGF-β1
	M2: Tolerance: CTLA4	Anti-CTLA4
	M4: Counterattack: DcR3	Anti-DcR3
	M5: Impaired antigen presentation	DC vaccine
**Cluster 7 (Luminal B, IDC)**	M1: Immunosuppression: TGF-β1	Anti-TGF-β1
	M2: Tolerance: CTLA4, IFN-γ	Anti-CTLA4; anti-IFN-γ*

*: require further investigation

¥: These include BIRC3, TRAF1, TNFAIP3 and TRAILR4; IDC: invasive ductal carcinoma; ILC: invasive lobular carcinoma.

DcR3 and TGF-β1 were shown to work in concert to induce epithelial to mesenchymal transitioning in colorectal cancer [22]. Thus, it is possible that these molecules work together in breast cancer as well to aid in tumor progression and immune evasion and thus, serve as potential immunotherapy targets.

### ILC and IDC show distinctive evasion combinations

Forty-two percent of TCGA’s ILC were significantly associated with clusters 3 and 6 while only 28.5% of IDC patients were significantly associated with clusters 2, 5 and 7 (Table 2). Although ILC is less common than IDC, studies have suggested that overall long-term outcomes of patients with ILC may be worse than those with stage-matched IDC and ILC patients are less responsive to neoadjuvant chemotherapy [10,11]. An obvious distinction between the 2 subgroups was that IDC clusters have fewer combinations of mechanisms compared to ILC clusters and the counterattack via DcR3 (M4) was exclusive to ILC. Since certain chemotherapy treatments have immune-stimulating properties, they can help sensitize the immune response by promoting antigen presentation and tumor sensitization to T-cell mediated killing by Treg depletion [8,23]. Thus, M1, M2, and M5 could be diminished by chemotherapy. This might be the reason why IDC derives greater benefit from chemotherapy compared to ILC which also evades via DcR3 and induces T cell apoptosis. However, this hypothesis requires further validation.

### TNBC falls into 2 evasion clusters

TNBC was significantly associated with clusters 1 and 2, Luminal A with clusters 3 and 4, and Luminal B with clusters 5 and 7 (Table 1). TNBC patients were mostly distributed between cluster 1 which was identified with 4 different evasion mechanisms (47%) and cluster 2 where evasion is mainly driven by impaired antigen presentation (42%). Thus, there are 2 immune evasion subgroups for TNBC: one with only impaired antigen presentation and another with a combination of 4 evasion mechanisms.

Although TNBC is associated with poor prognosis, certain patients seemed to respond well to anthracycline-based chemotherapies [8]. Previous studies have shown that objective complete response was significantly associated with immune modules only in luminal breast cancer subgroups and that the tumor-infiltrating lymphocytes in TNBC had no significant interaction with paclitaxel plus non-pegylated liposomal doxorubicin-based therapies in neoadjuvant settings [9,24]. In postneoadjuvant setting, tumor-infiltrating lymphocytes were associated with better prognosis in TNBC [25]. Since chemotherapy may induce immunogenic cell death [23], it is possible that TNBC patients falling into cluster 2 can overcome the impaired antigen presentation after chemotherapy-induced sensitization, resulting in the recruitment of TIL. However, more investigations are required to validate this possibility.

Furthermore, cluster 1 immune gene expression seems to overlap with the immunomodulatory subtype of TNBC, which is characterized by upregulated expression of genes involved in T cell function, interferon response, and antigen presentation [26].

### Identification of biomarkers for the immune evasion clusters

The discovery of seven immune evasion groups for breast cancer helps provide guidance for the choice of treatment, if we can identify for any given patient the cluster they belong to and hence, their evasion mechanisms. Using the molecules directly related to the immune evasion mechanisms, such as PD-L1, CTLA4, etc., may not be optimal especially that our results showed that evasion mostly occurs by a combination of several mechanisms and several molecules. Instead, we generated a decision tree and identified 12 biomarkers along with their gene expression thresholds (Fig 4). Interestingly, the identified immune evasion genes were not all part of this set of putative biomarkers, which indicates that evasion genes may not be powerful in terms of identifying patients’ evasion mechanisms. S8 File summarizes the rationale for using classifier genes for each cluster and their corresponding functions.

## Discussion

Tumors evade immune surveillance using 6 different mechanisms, which may occur simultaneously in the same tumor (Fig 1). Thus, there are 63 possible combinations of mechanisms in BRCA. This heterogeneity even at the level of a single cancer hallmark explains the challenge of identifying effective treatments.

Using the sequential biclustering method on BRCA RNA-seq data from TCGA, we identified seven clusters of BRCA patients with different evasion mechanisms and combinations of mechanisms. The mechanisms of evasion were determined based on the expression of immune evasion genes and the genes involved in the cancer-immunity cycle. To make it easier to identify patients’ evasion group and thus, the choice of immunotherapy, we narrowed down the list of immune-related genes to 12 biomarkers using a classification tree algorithm. Fitting a single tree may not be able to achieve the maximum degree of accuracy, however, the classification tree was used due to its intuitive output with straightforward clinical interpretations. To further improve the classification accuracy, we applied the random forest model to the same data set (S9 File).

Our results indicate that there are different combinations of evasion mechanisms involved in breast cancer. We found that 77.4% of the clustered patients evade with TGF-β1-induced immunosuppression and 57.7% with a DcR3-induced counterattack. All clustered patients with upregulated DcR3 expression had TGF-β1 upregulated as well. Thus, targeting TGF-β1 alone or with DcR3 may be promising for breast cancer treatment. The immunosuppressive nature of TGF-β has been well studied. It was shown that TGF-β disrupts antigen presentation and T cell activation, induces a Treg transformation from naïve T cells, and causes epithelial to mesenchymal transitioning [27–31]. The blockade of TGF-β in colon cancer unleashed a potent and endured cytotoxic T-cell response against cancer cells, inhibited metastases, and rendered metastatic colon cancer more susceptible to anti-PD-1-PD-L1 therapy [32]. DcR3 was shown to inhibit cytotoxicity against tumor cells and its expression was positively associated with cancer progression, angiogenesis, and metastasis [33,34]. Furthermore, DcR3 was suggested as a prognostic factor for early tumor detection and a predictor of recurrence after resection in breast cancer, specifically [35,36].

Examining the invasive breast cancer subgroup, we found that, compared to IDC, ILC-associated clusters had an exclusive upregulation of DcR3. This led us to hypothesize that the reason IDC benefits more from chemotherapy is that the latter helps diminish evasion by aiding in antigen presentation and killing Tregs [8,23]. However, the upregulated expression of DcR3 in the ILC-enriched clusters results in further cytotoxic T cell death. Our results contradict with a previous study on a northeastern Chinese population where they showed increased expression of DcR3 in IDC [36], however, our population is representative of a large racial and ethnic population.

Due to the higher immune system-tumor interactions involved, TNBC and HER2 were thought to be more immunogenic than Luminal A [37]. However, our results showed that TNBC patients split into two groups: the highly immunogenic cluster1 (TNBC: 51/105) and the less immunogenic (low leukocyte infiltration) cluster 2 (TNBC: 45/105) (Table 1). Based on our results, we hypothesized that cluster 1 TNBCs correspond to the immunomodulatory TNBC subtype and that cluster 2 TNBC’s treatment with chemotherapy may trigger an immune response [23,26,38]. Furthermore, the comparison between the different clusters showed that only cluster 1 was significantly associated with a higher proliferation gene expression signature (S10 File). However, given the strong immune response in cluster 1, this may be caused by the highly proliferating immune cells recruited to the tumor microenvironment rather than cancer cells’ proliferation.

Currently, there are no clinical trials on DcR3 however, there are several ongoing in breast cancer for blockading CTLA4, TGF-β, PD-1/PD-L1, CLTA4 and PD-1, and chimeric antigen receptor T-cell therapy (CAR-T). The list is summarized in S1 File. The response rate of blockading PD-1/PD-L1 in breast cancer patients has been tested in several hormone receptor subtypes. The overall response rate to PD-1 blockade in PD-L1 positive patients with advanced TNBC BRCA was 18.5% [39] and 12% in ER+ and HER 2- advanced BRCA [40]. The overall response rate to anti-PD-L1 was 24% in TNBC [41], and only 3% in metastatic BRCA—62.5% of whom were PD-L1+ [42]. In this latter study patients with TNBC experienced an overall response rate of 5.2% [42]. No drugs targeting CTLA4 alone are currently in clinical trial. Combining anti-PD-1 anti-CTLA4 in eighteen patients with refractory metastatic BRCA resulted in an overall response rate of 17%: 0% for the 11 ER+ patients and 43% for the 7 TNBC patients [43]. However, the small TNBC sample size makes the results inconclusive. Overall, these results show a poor overall response rate when the treatment criteria are based on breast cancer receptor subtypes and the expression of PD-1/PD-L1 and CTLA4, stressing the need for new criteria and more reliable biomarkers. Our results showed that there is no one hormone receptor subtype that falls into one immune evasion cluster, rendering receptor subtyping a weak guide to the choice of immunotherapy. We also showed that the expression of PD-1 and CTLA4 is not a good biomarker for immunotherapy and hence the need for more reliable biomarkers.

Despite the large breast cancer sample size in TCGA, the absence of matched normal samples for about 90% of the breast cancer patients prevented us from taking individual genetic variation into account in this study. Moreover, the TCGA normal samples are not exactly normal but non-malignant adjacent samples.

## Supporting information

S1 File Potential immunotherapies for breast cancer patients. This table summarizes potential treatment methods, drug names, FDA status, and whether or not it is in a clinical trial for breast cancer.
(XLSX)

S2 File Immune gene lists and the number of patients and genes in the identified clusters. (XLSX)

S3 File Consistency measure and cross validated error rates by classification tree model. (XLSX)

S4 File T-test results for tumor versus tumor and tumor versus normal comparisons for the 7 clusters. (XLSX)

S5 File Immune pathways generated by Pathview package. (ZIP)

S6 File Categorized gene expression results into the different steps of the cancer-immunity cycle and different immune evasion mechanisms. (XLSX)

S7 File A detailed explanation of the evasion mechanisms in each cluster. (DOCX)

S8 File Identified biomarkers using the classification tree. (XLSX)

S9 File Random forest results. (DOCX)

S10 File Enrichment analysis for the proliferation GO term. (XLSX)
