# Supplementary figures and images for: Subgrouping breast cancer patients based on immune evasion mechanisms unravels a high involvement of transforming growth factor-beta and decoy receptor 3
Source: PLoS One. 2018 Dec 4;13(12):e0207799. doi: 10.1371/journal.pone.0207799 (PMC6279052; doi:10.1371/journal.pone.0207799)

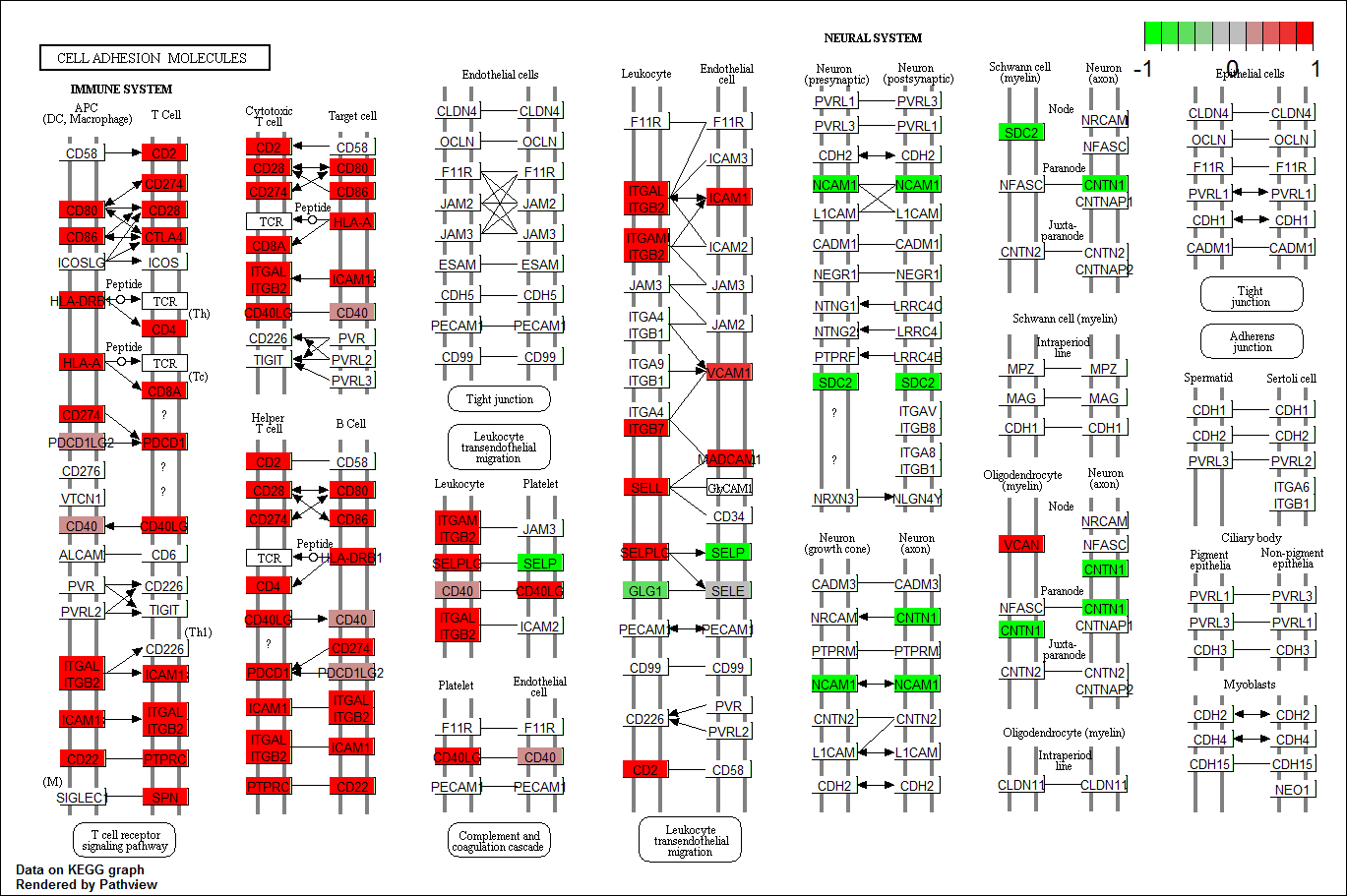

Supplement: S5 File — (ZIP) [file pone.0207799.s005.zip › S5_File/hsa04514.Tum_Nor1.png]

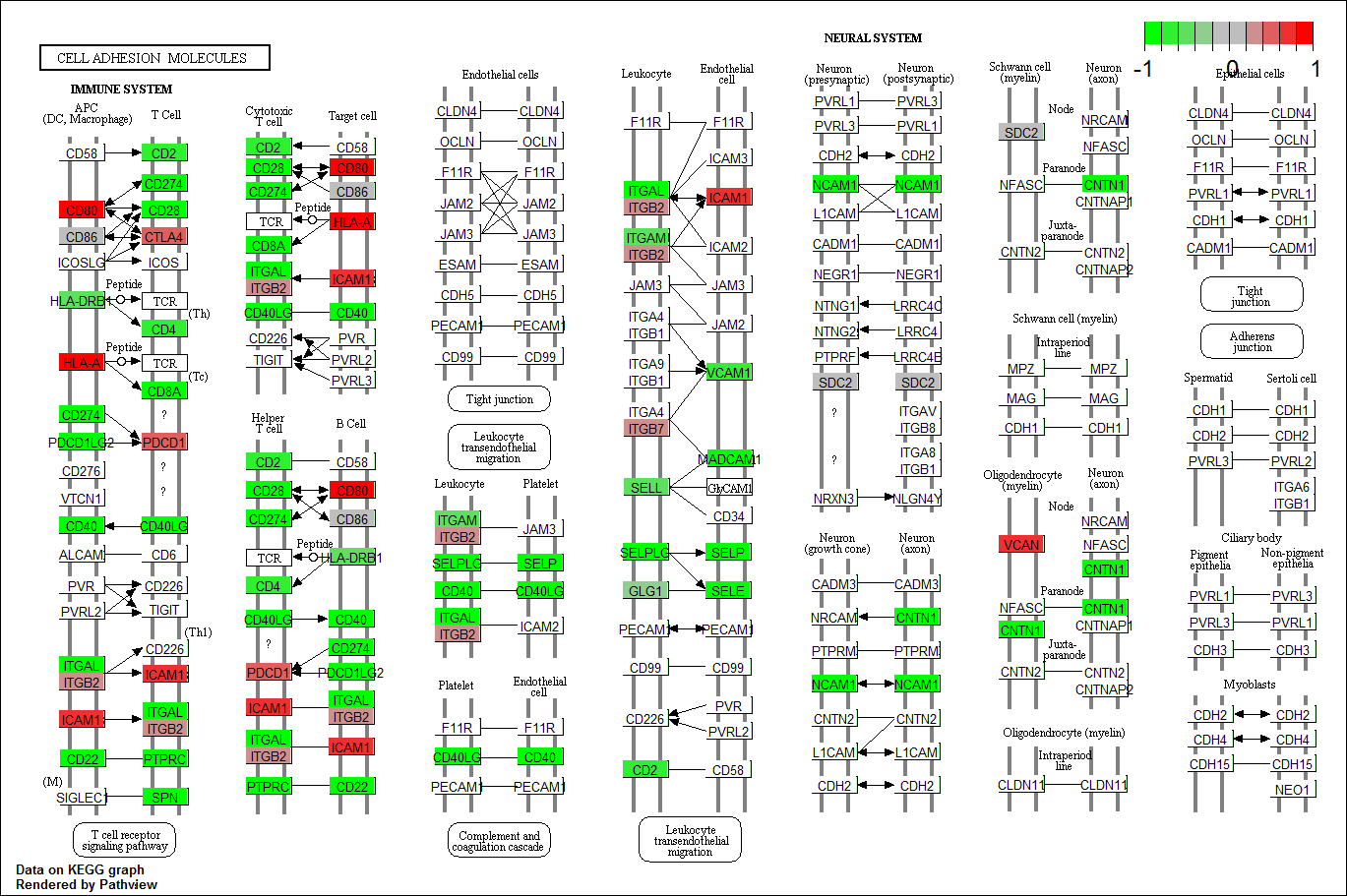

Supplement: S5 File — (ZIP) [file pone.0207799.s005.zip › S5_File/hsa04514.Tum_Nor2.png]

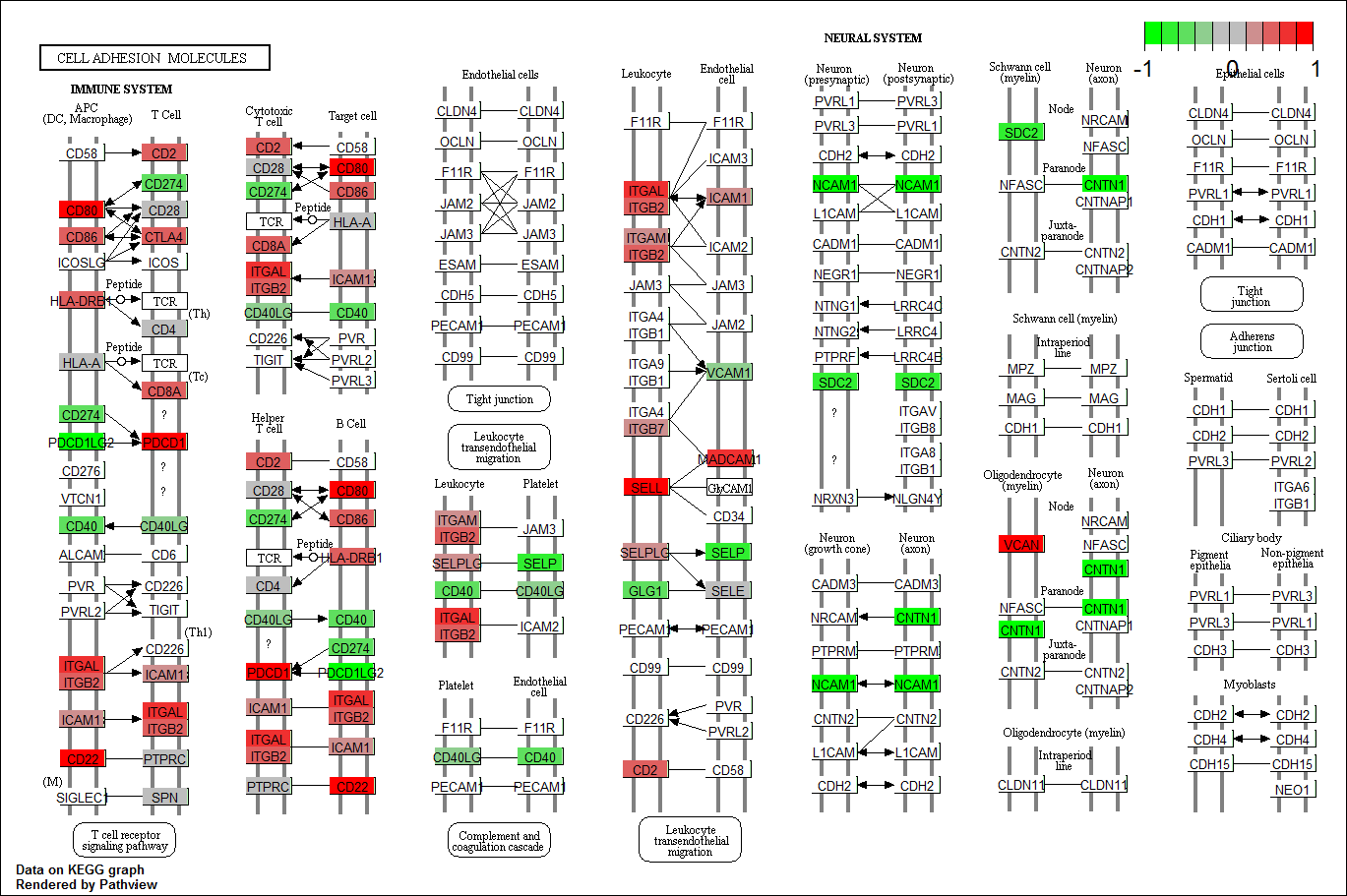

Supplement: S5 File — (ZIP) [file pone.0207799.s005.zip › S5_File/hsa04514.Tum_Nor3.png]

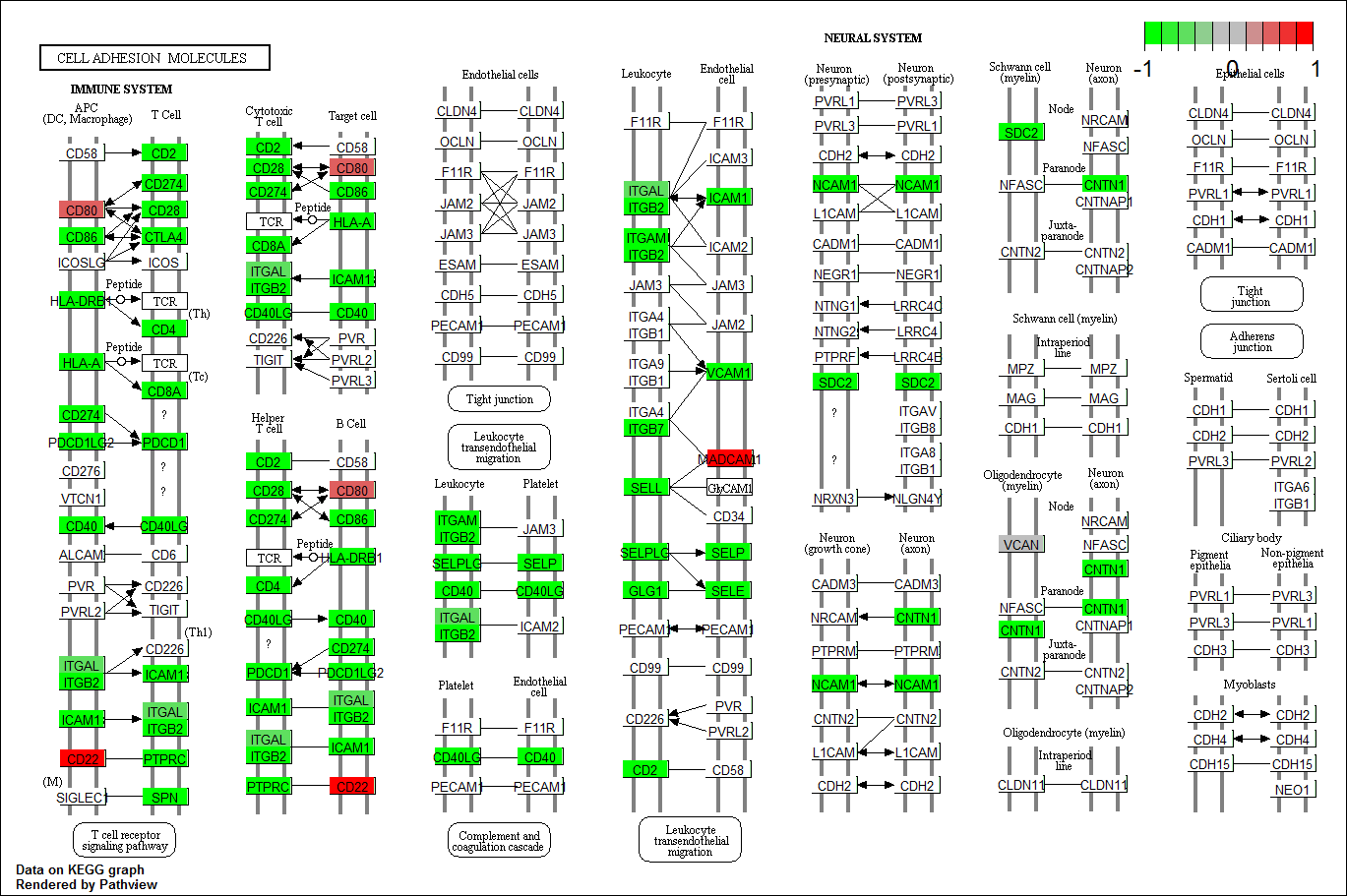

Supplement: S5 File — (ZIP) [file pone.0207799.s005.zip › S5_File/hsa04514.Tum_Nor4.png]

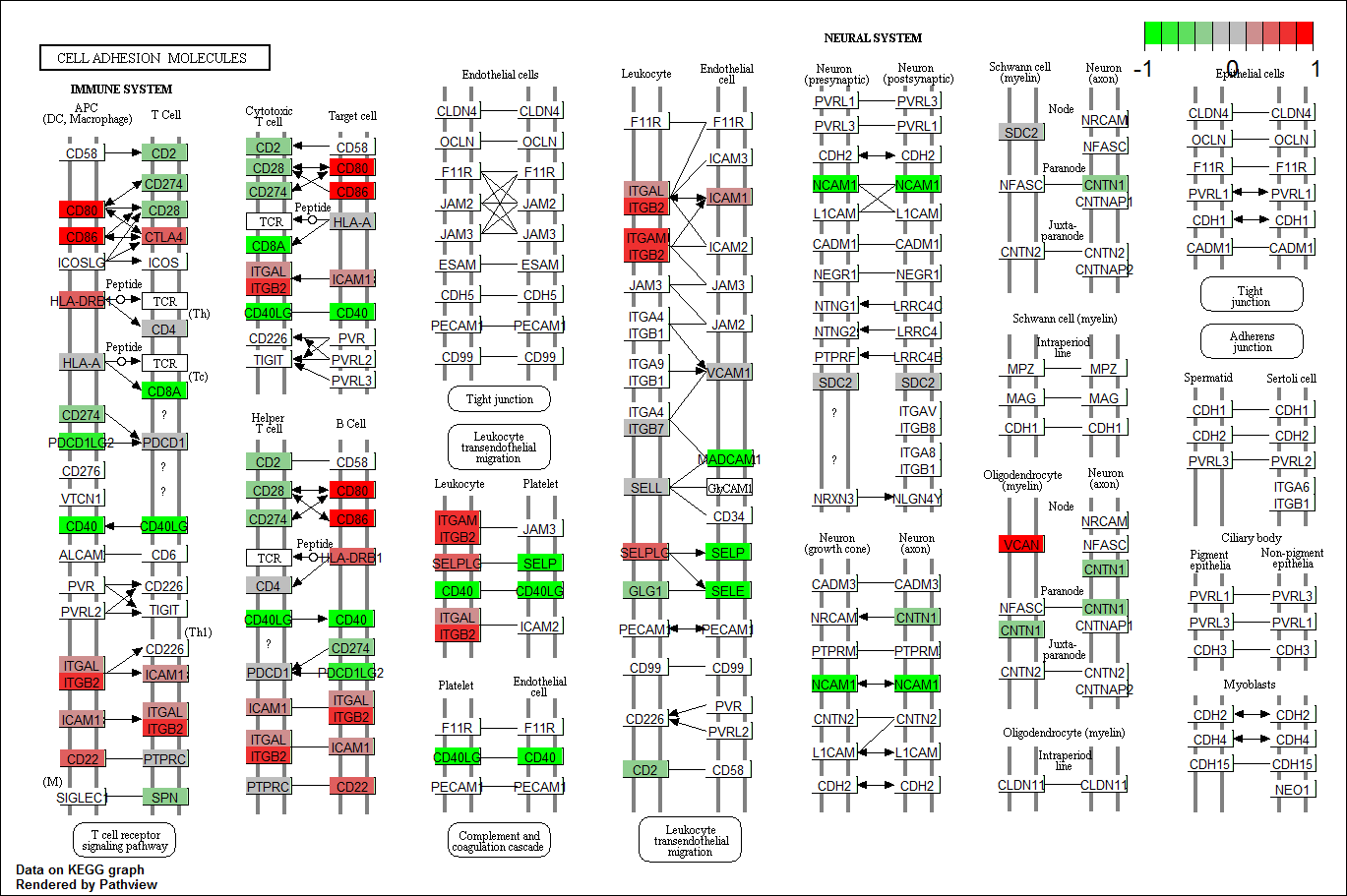

Supplement: S5 File — (ZIP) [file pone.0207799.s005.zip › S5_File/hsa04514.Tum_Nor5.png]

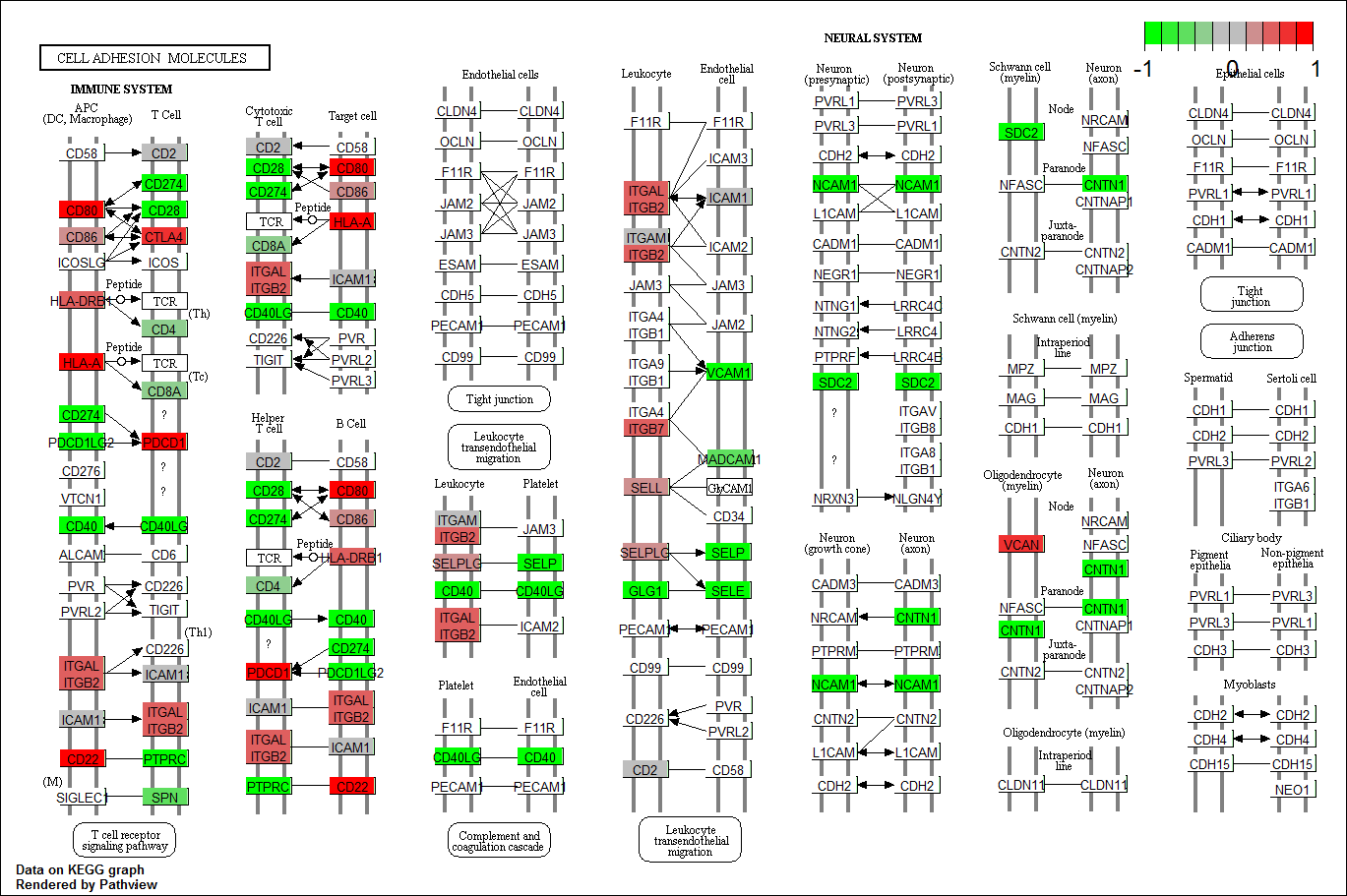

Supplement: S5 File — (ZIP) [file pone.0207799.s005.zip › S5_File/hsa04514.Tum_Nor6.png]

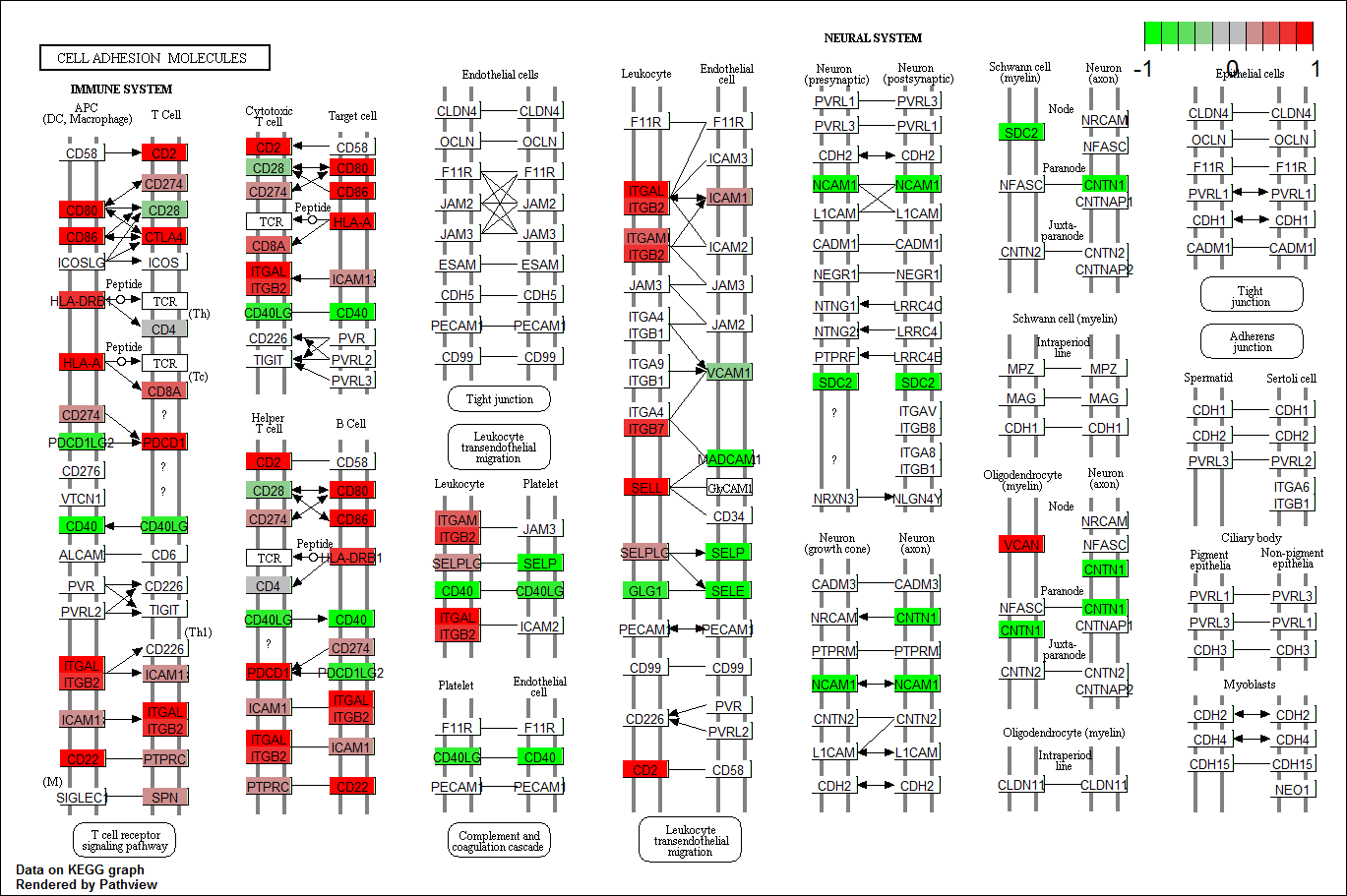

Supplement: S5 File — (ZIP) [file pone.0207799.s005.zip › S5_File/hsa04514.Tum_Nor7.png]

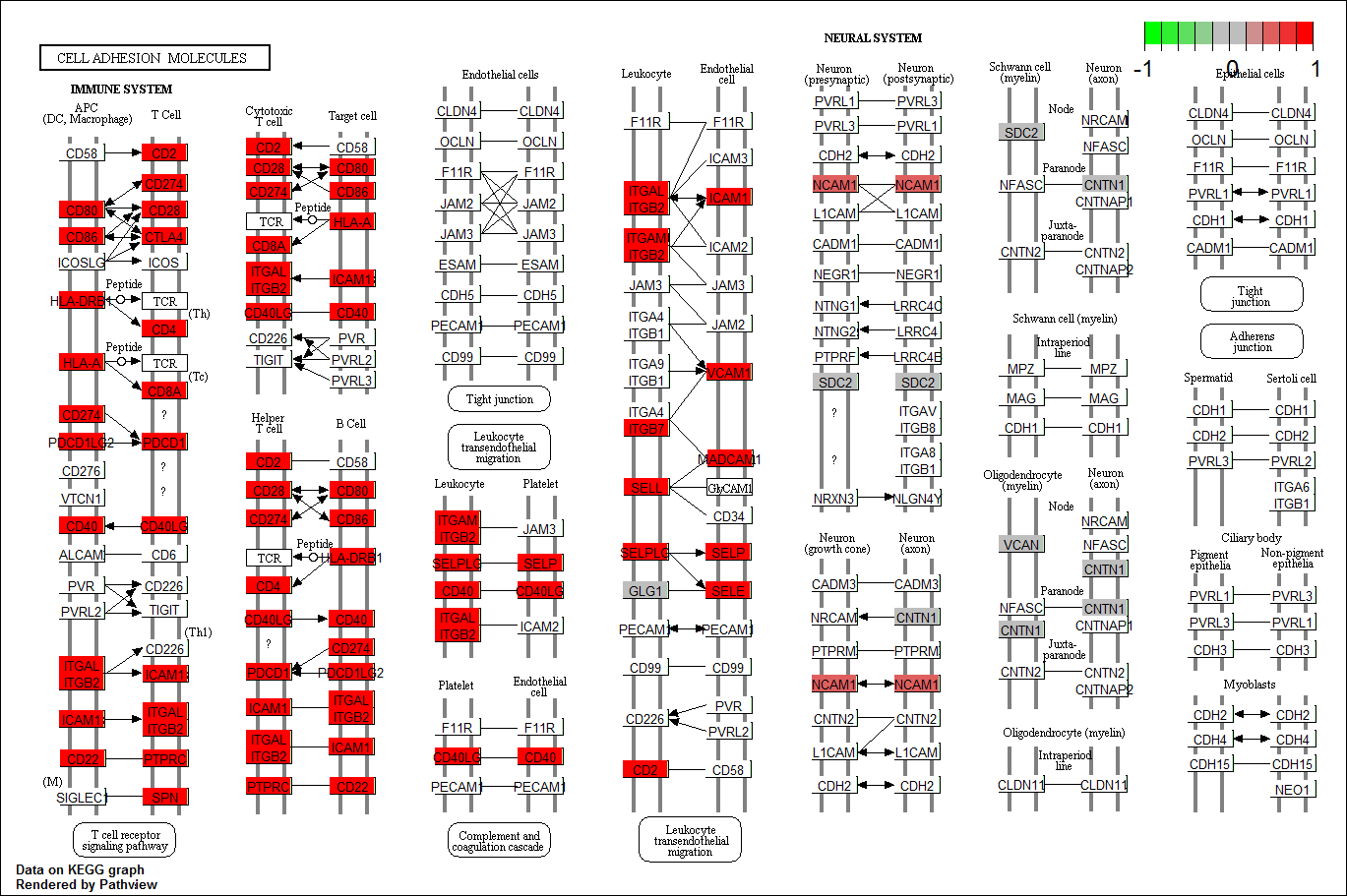

Supplement: S5 File — (ZIP) [file pone.0207799.s005.zip › S5_File/hsa04514.Tum_Tum1.png]

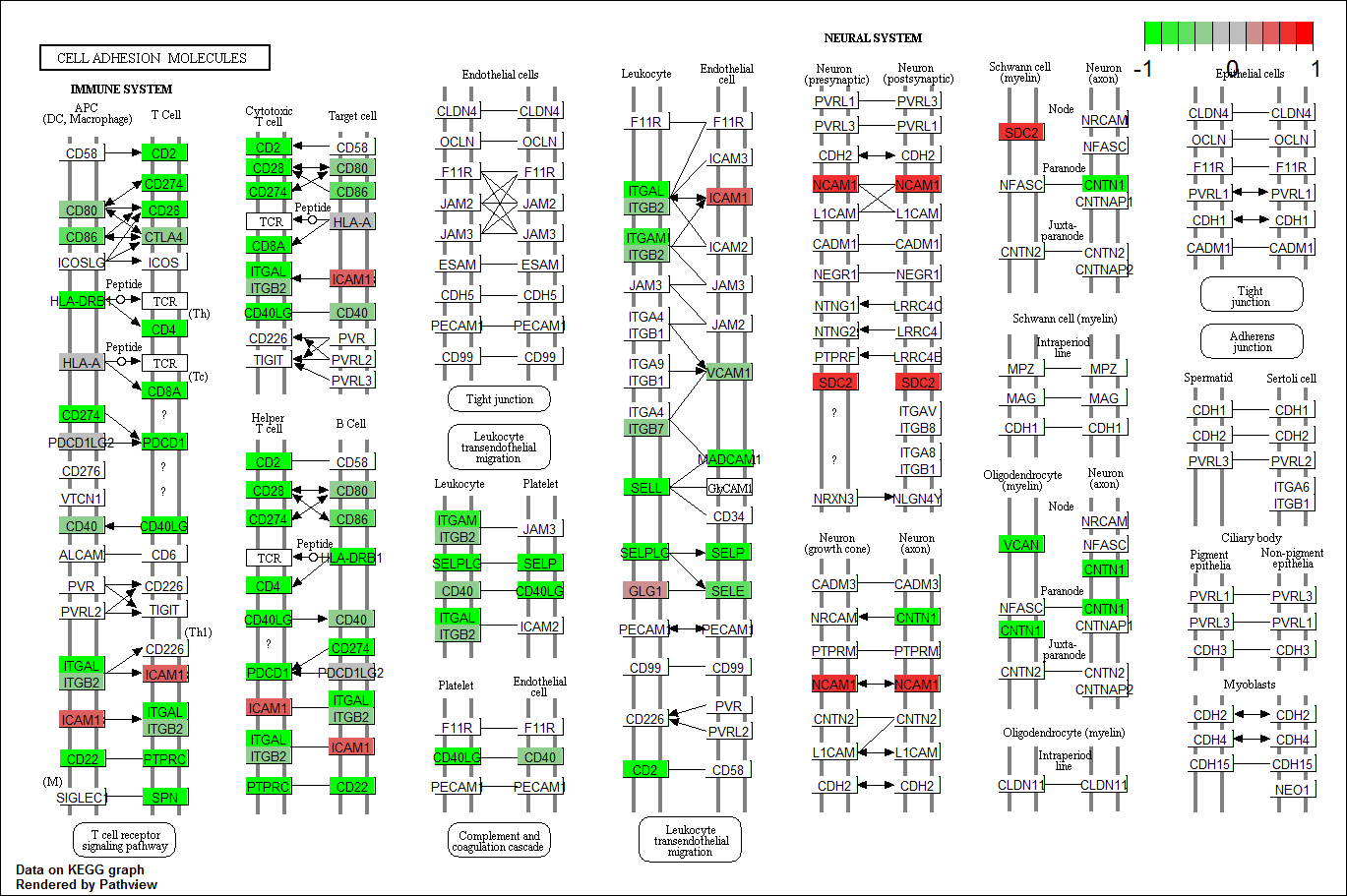

Supplement: S5 File — (ZIP) [file pone.0207799.s005.zip › S5_File/hsa04514.Tum_Tum2.png]

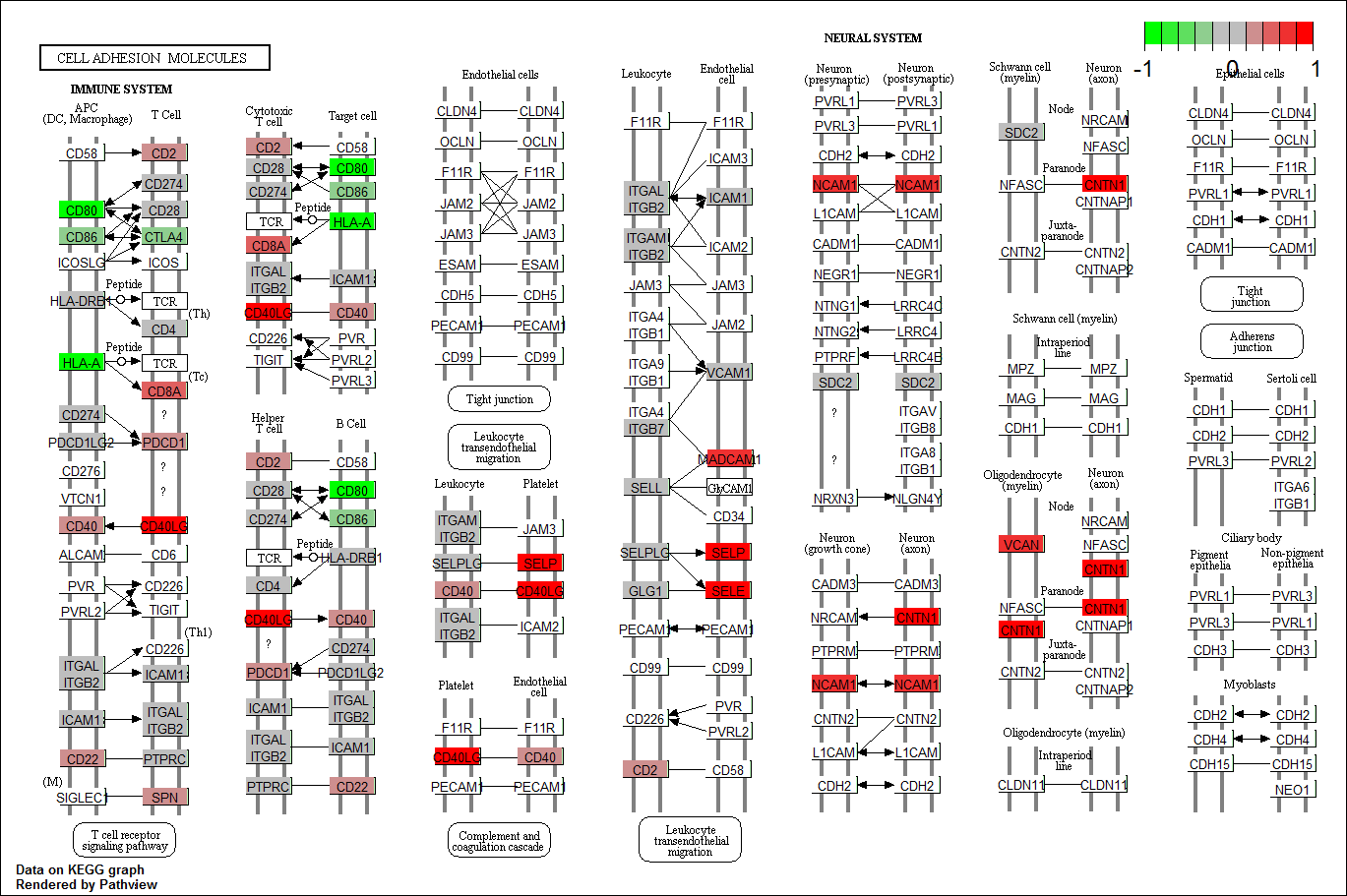

Supplement: S5 File — (ZIP) [file pone.0207799.s005.zip › S5_File/hsa04514.Tum_Tum3.png]

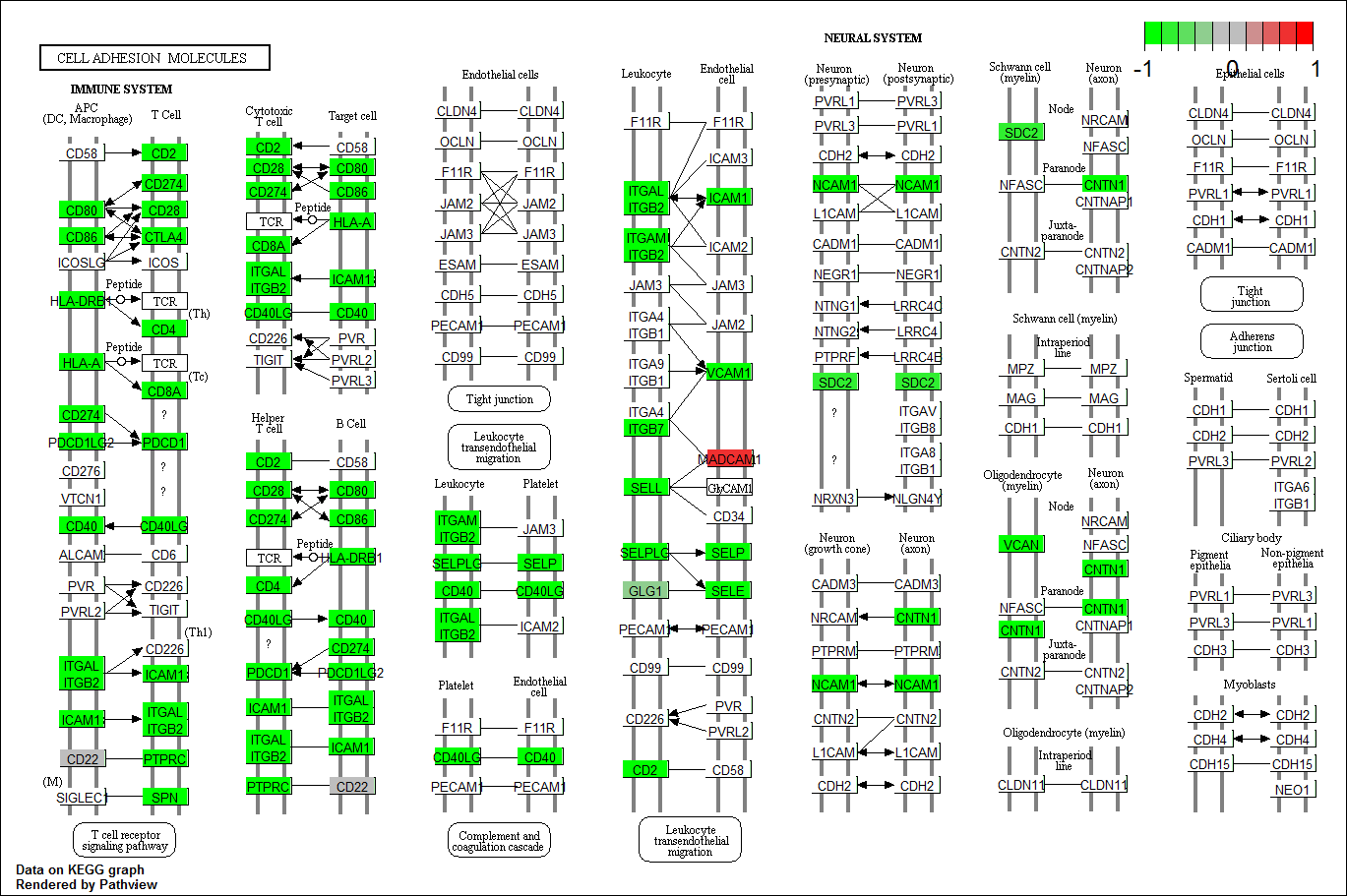

Supplement: S5 File — (ZIP) [file pone.0207799.s005.zip › S5_File/hsa04514.Tum_Tum4.png]

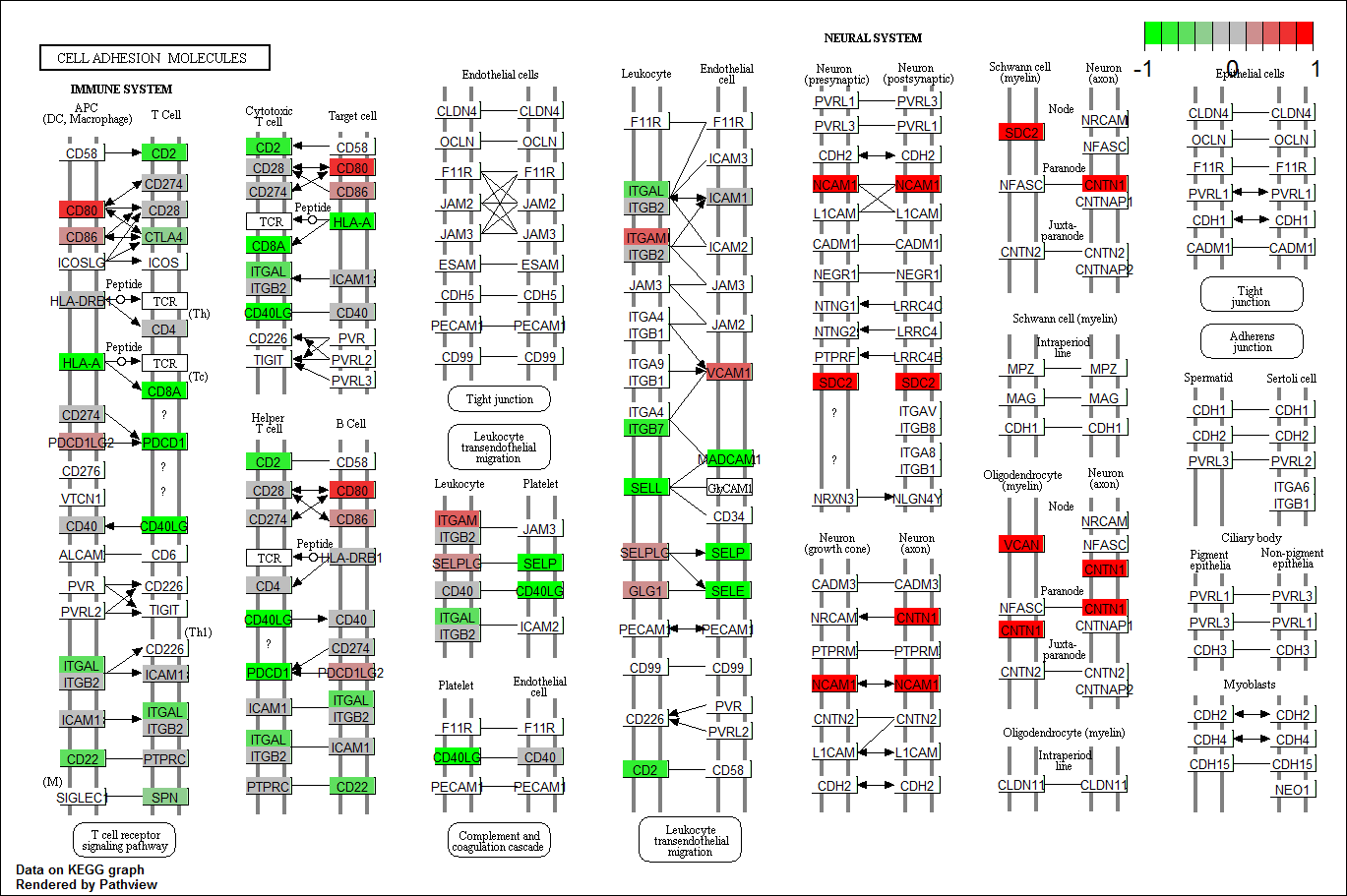

Supplement: S5 File — (ZIP) [file pone.0207799.s005.zip › S5_File/hsa04514.Tum_Tum5.png]

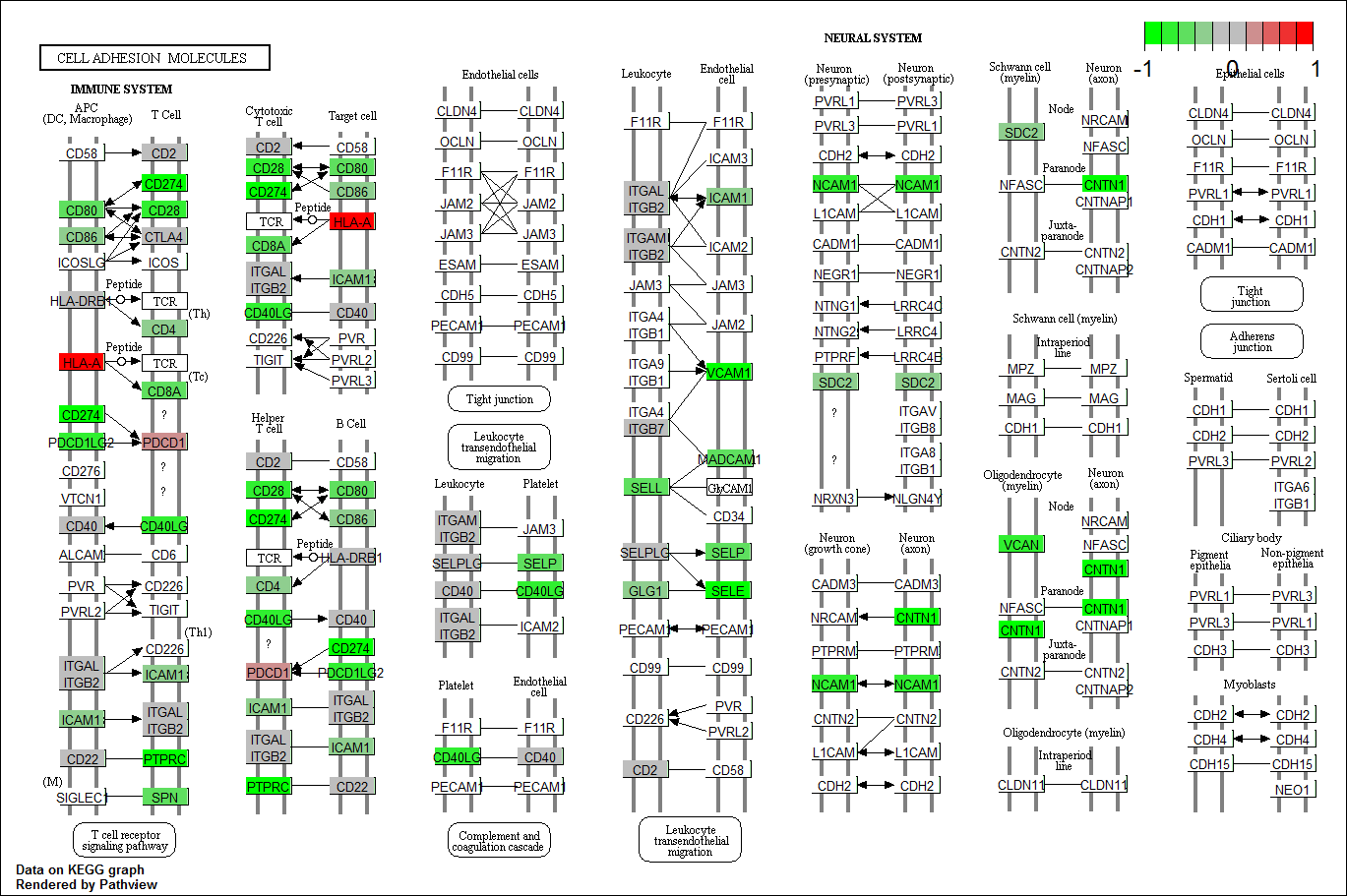

Supplement: S5 File — (ZIP) [file pone.0207799.s005.zip › S5_File/hsa04514.Tum_Tum6.png]

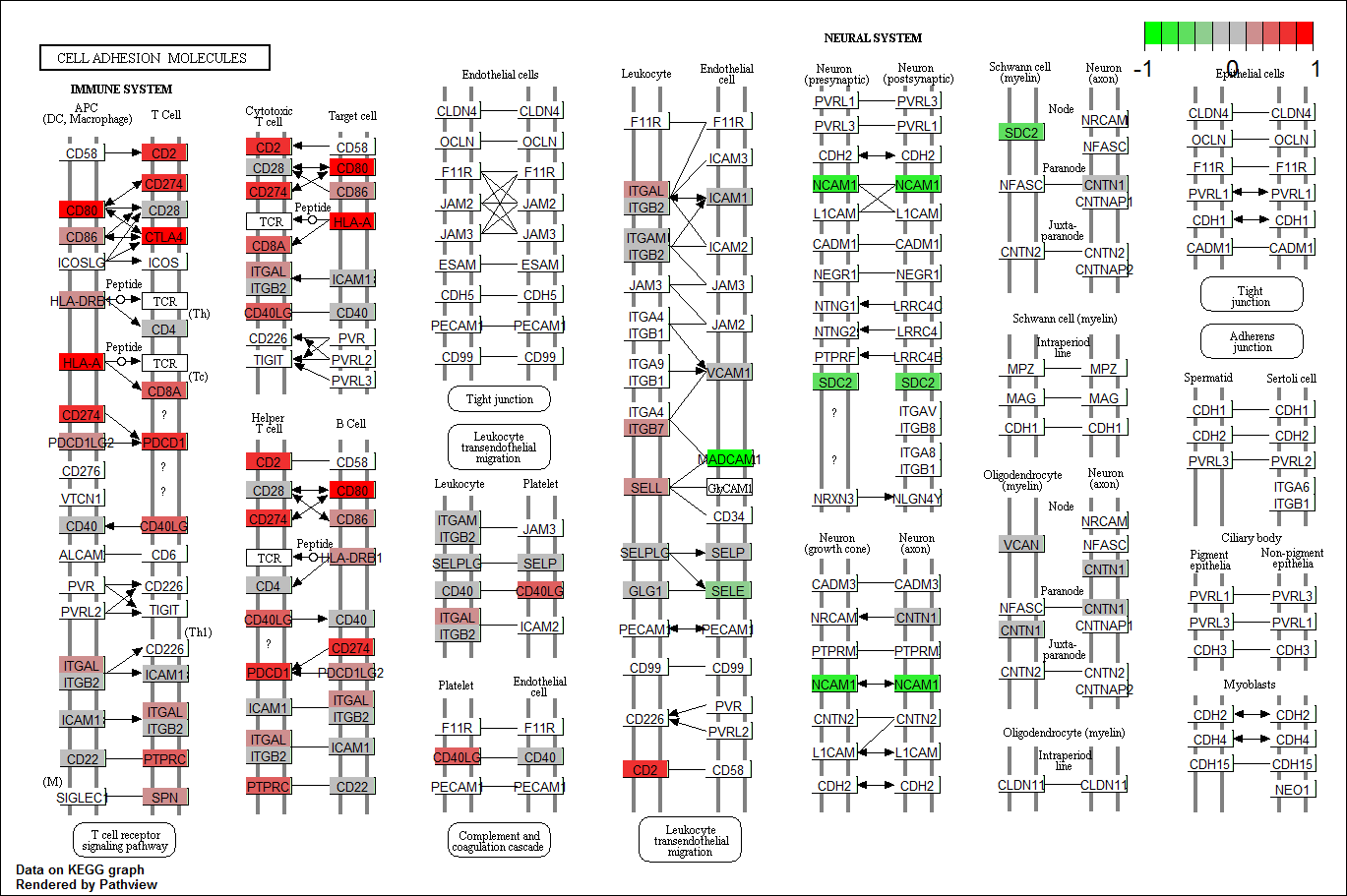

Supplement: S5 File — (ZIP) [file pone.0207799.s005.zip › S5_File/hsa04514.Tum_Tum7.png]

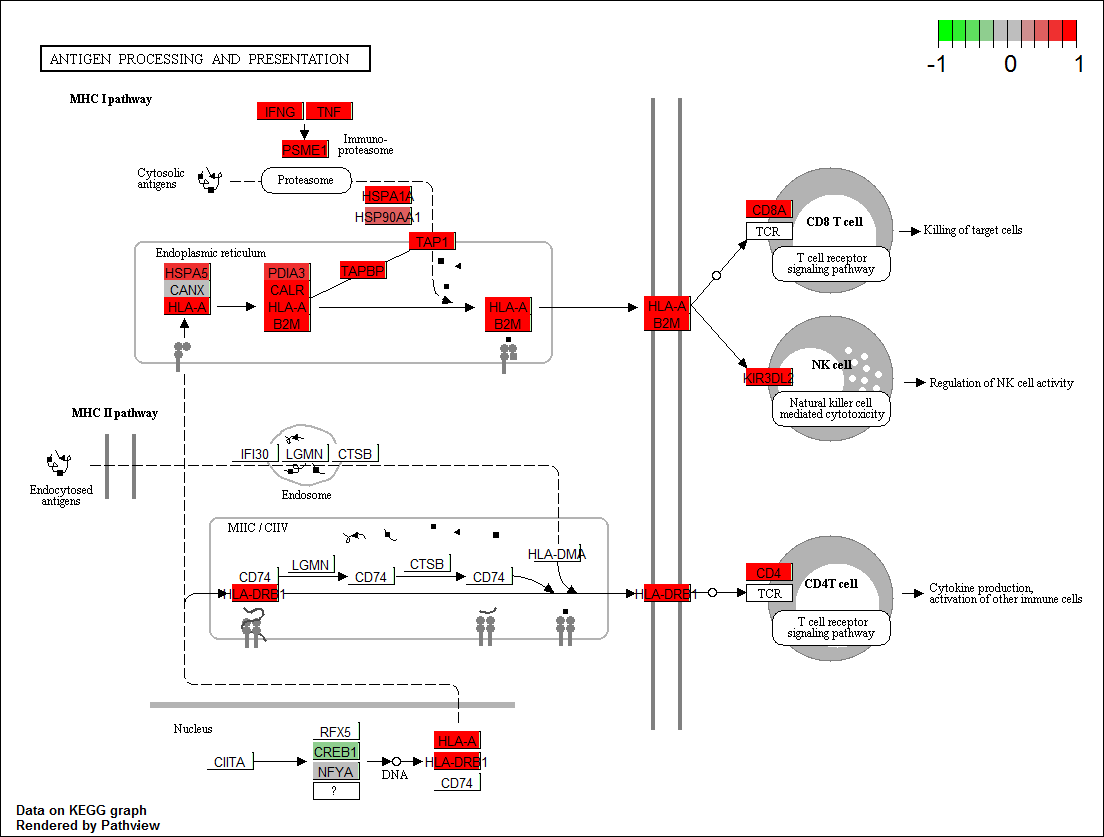

Supplement: S5 File — (ZIP) [file pone.0207799.s005.zip › S5_File/hsa04612.Tum_Nor1.png]

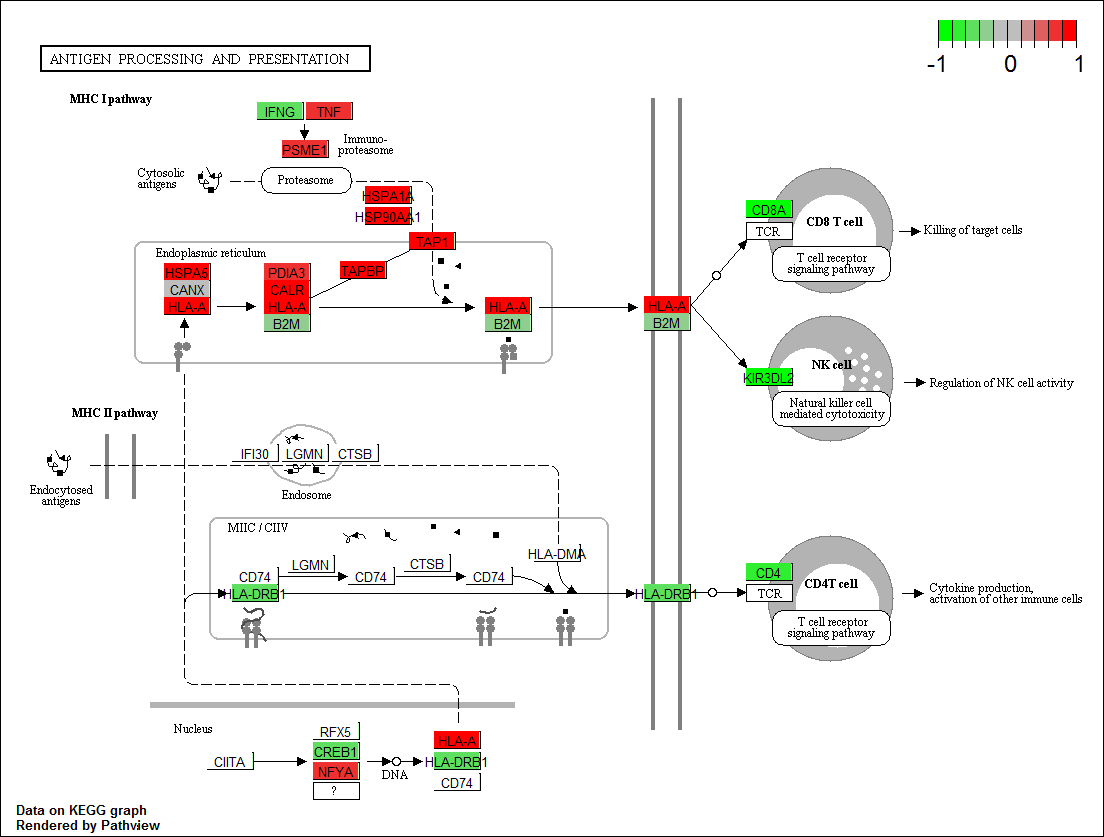

Supplement: S5 File — (ZIP) [file pone.0207799.s005.zip › S5_File/hsa04612.Tum_Nor2.png]

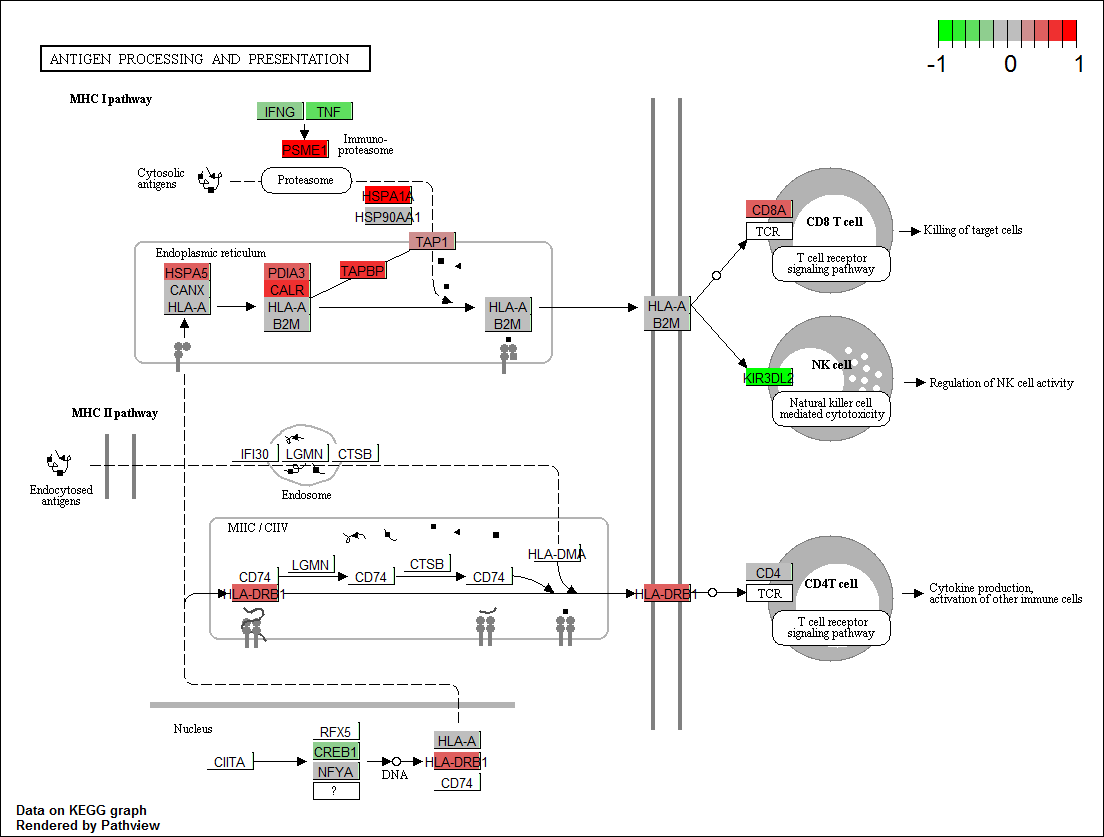

Supplement: S5 File — (ZIP) [file pone.0207799.s005.zip › S5_File/hsa04612.Tum_Nor3.png]

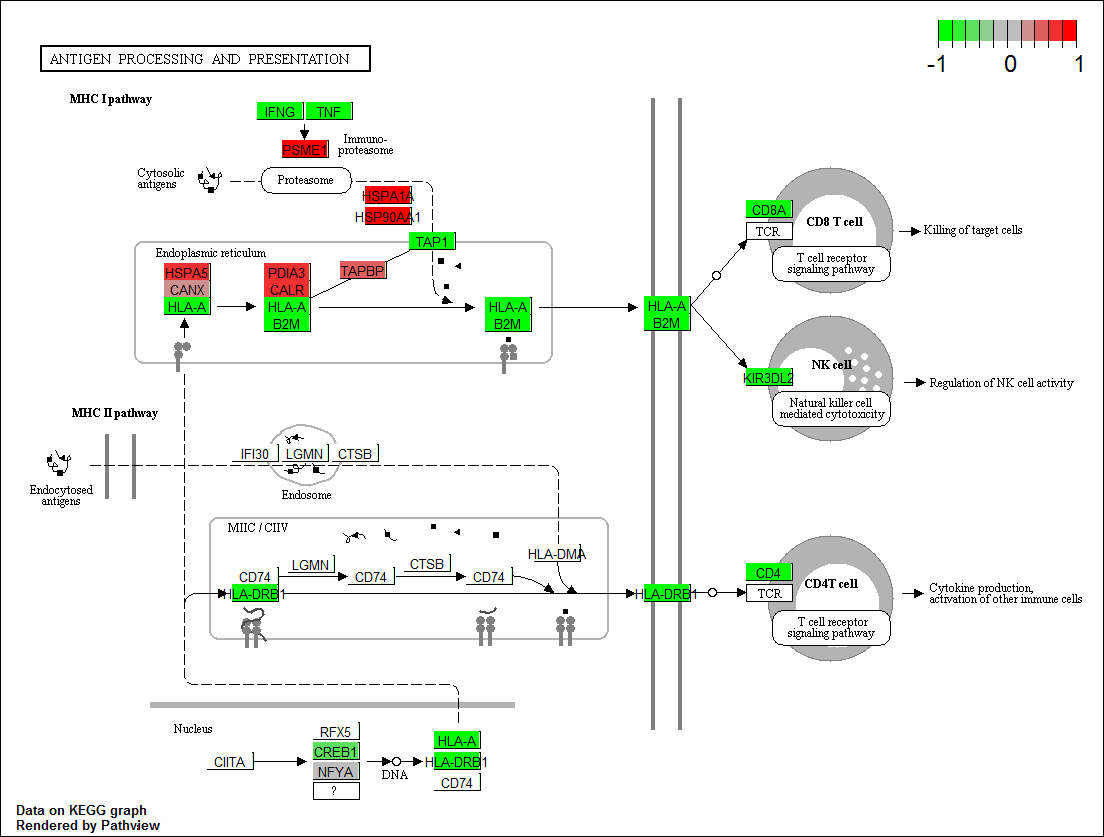

Supplement: S5 File — (ZIP) [file pone.0207799.s005.zip › S5_File/hsa04612.Tum_Nor4.png]

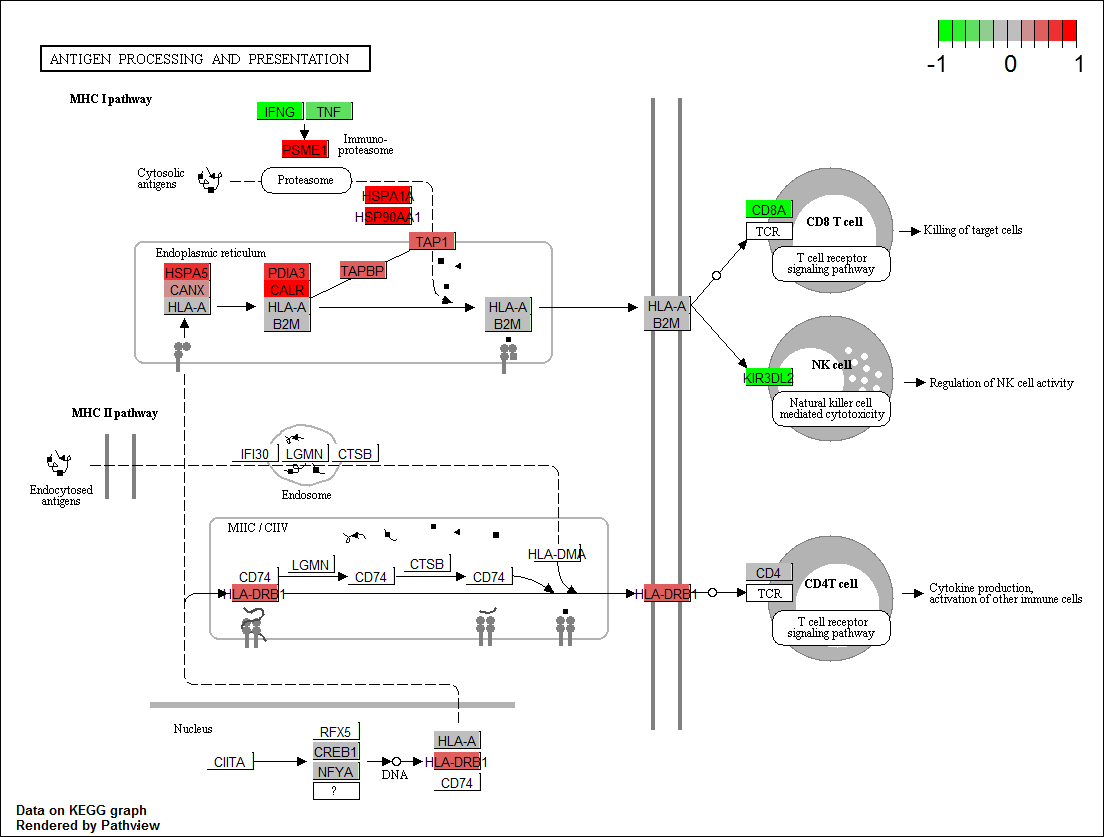

Supplement: S5 File — (ZIP) [file pone.0207799.s005.zip › S5_File/hsa04612.Tum_Nor5.png]

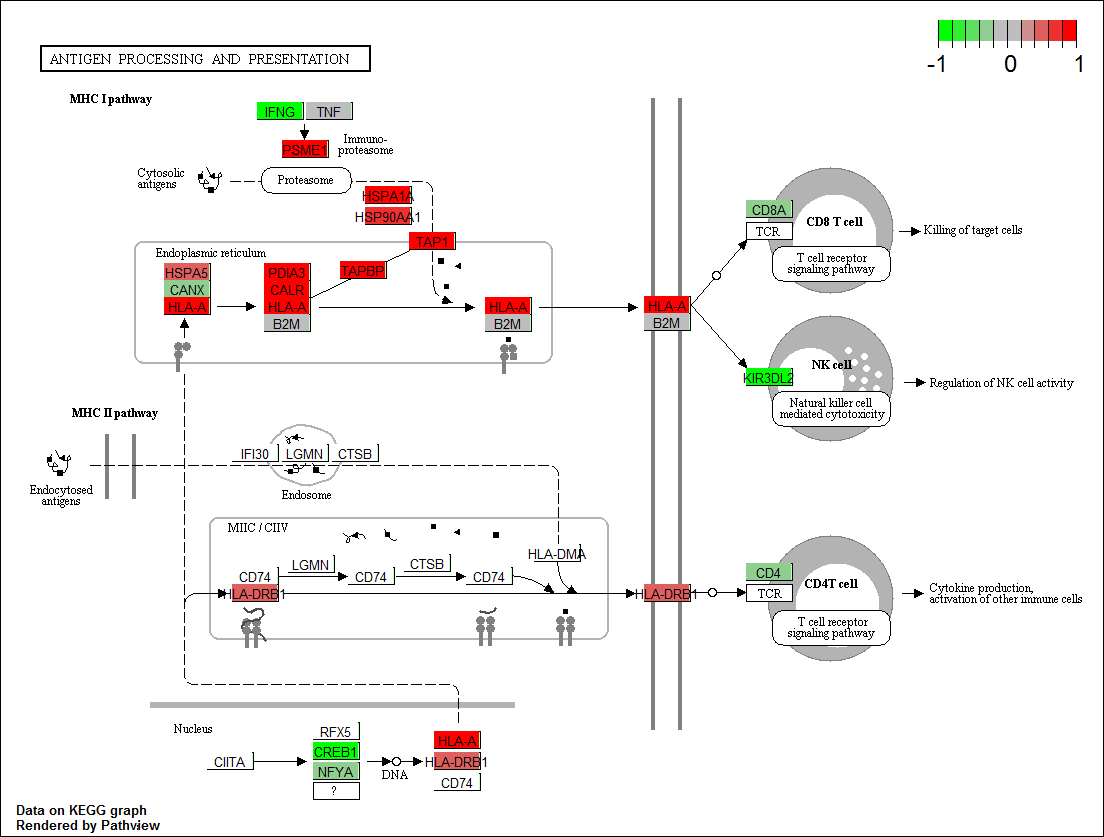

Supplement: S5 File — (ZIP) [file pone.0207799.s005.zip › S5_File/hsa04612.Tum_Nor6.png]

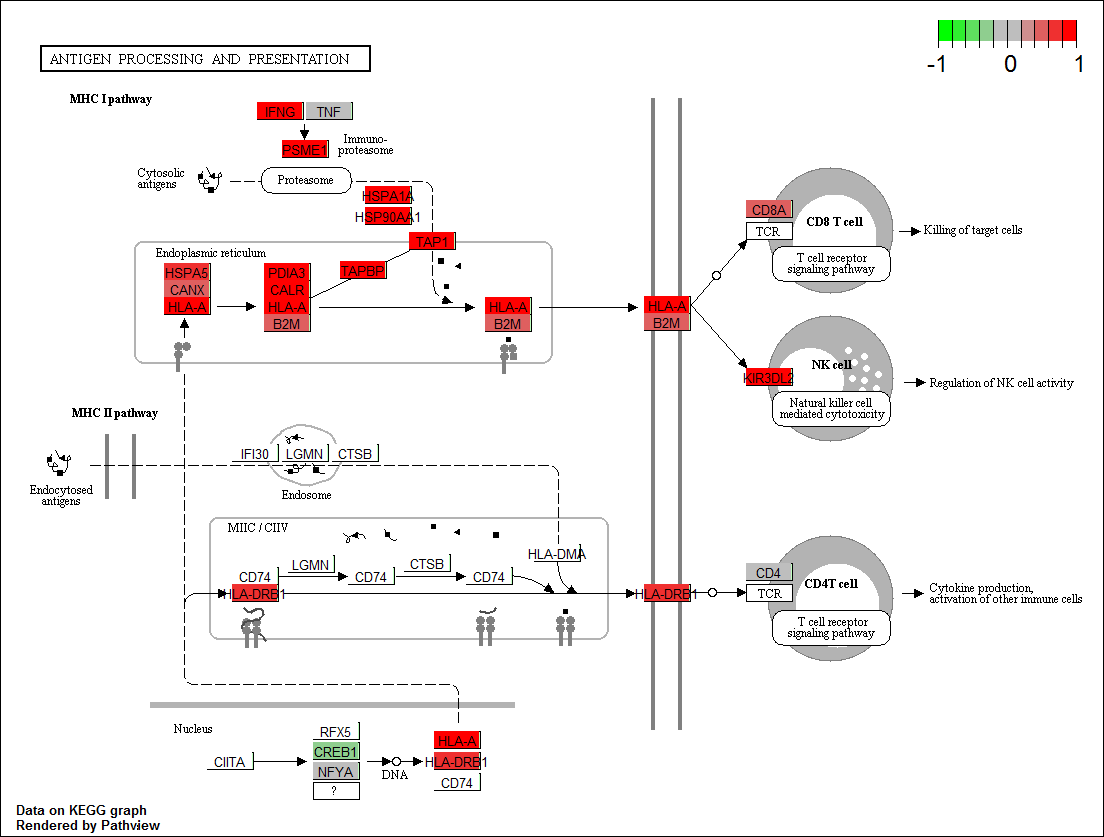

Supplement: S5 File — (ZIP) [file pone.0207799.s005.zip › S5_File/hsa04612.Tum_Nor7.png]

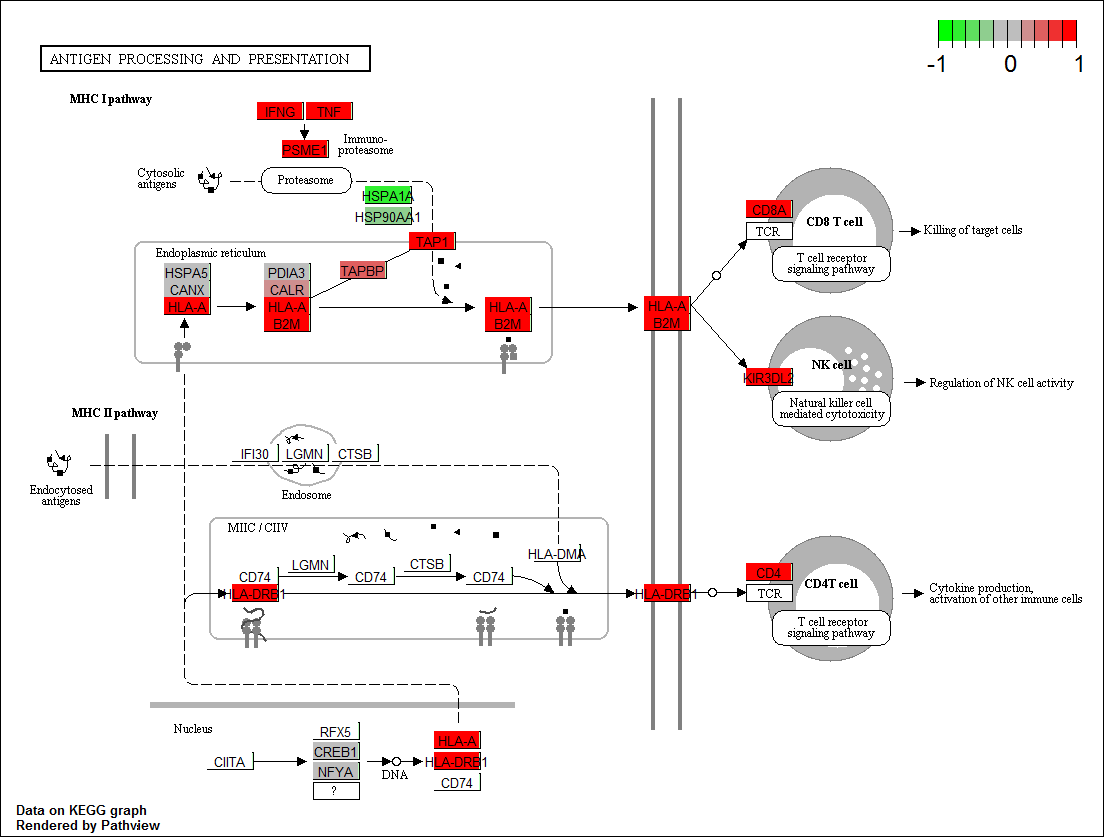

Supplement: S5 File — (ZIP) [file pone.0207799.s005.zip › S5_File/hsa04612.Tum_Tum1.png]

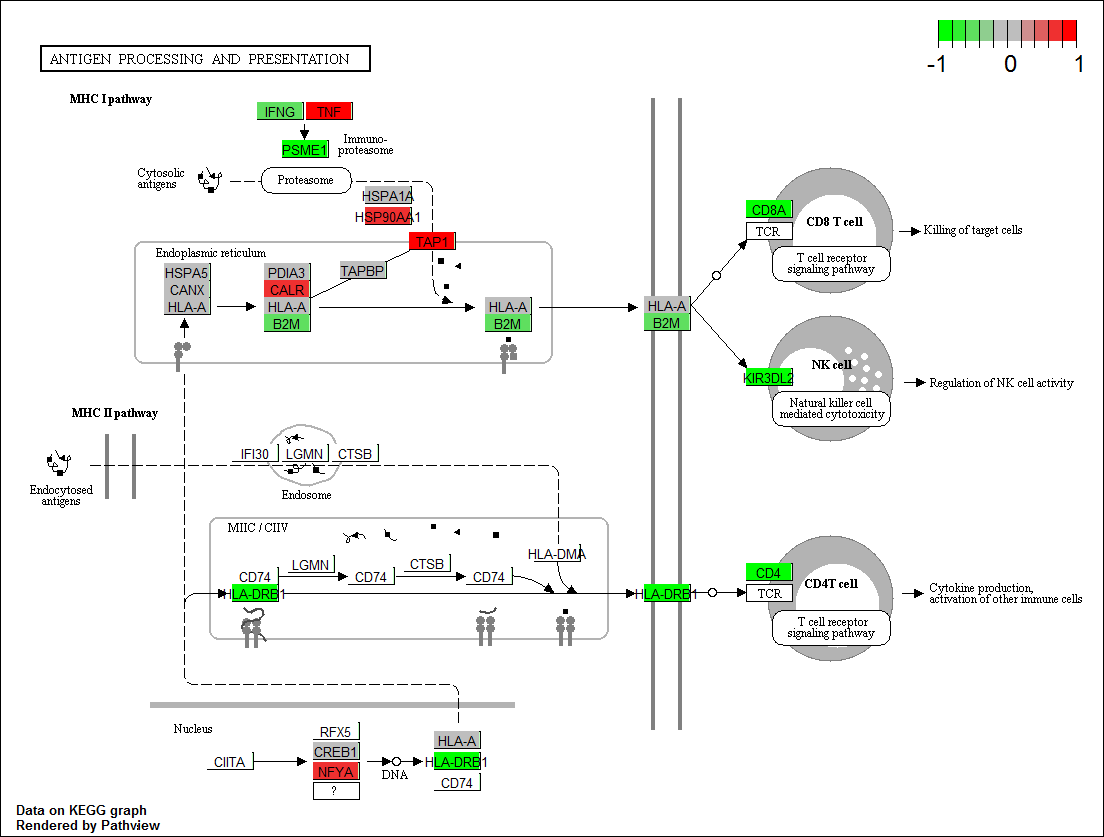

Supplement: S5 File — (ZIP) [file pone.0207799.s005.zip › S5_File/hsa04612.Tum_Tum2.png]

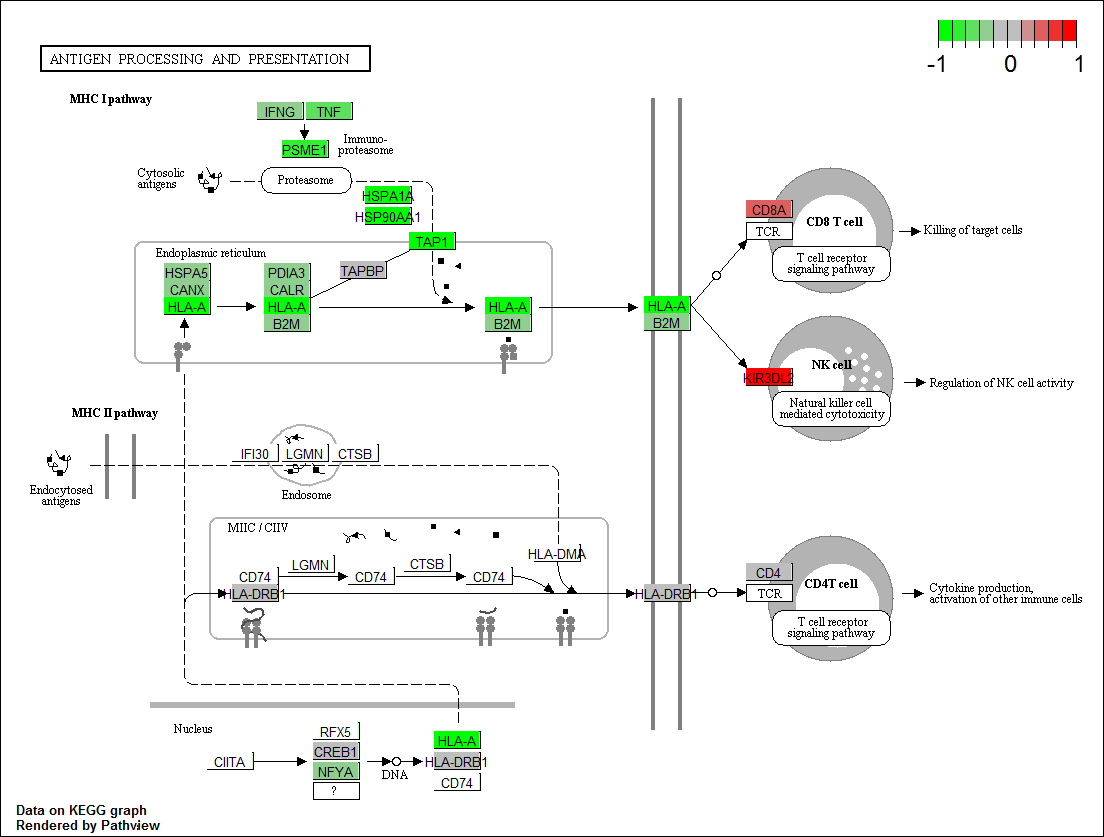

Supplement: S5 File — (ZIP) [file pone.0207799.s005.zip › S5_File/hsa04612.Tum_Tum3.png]

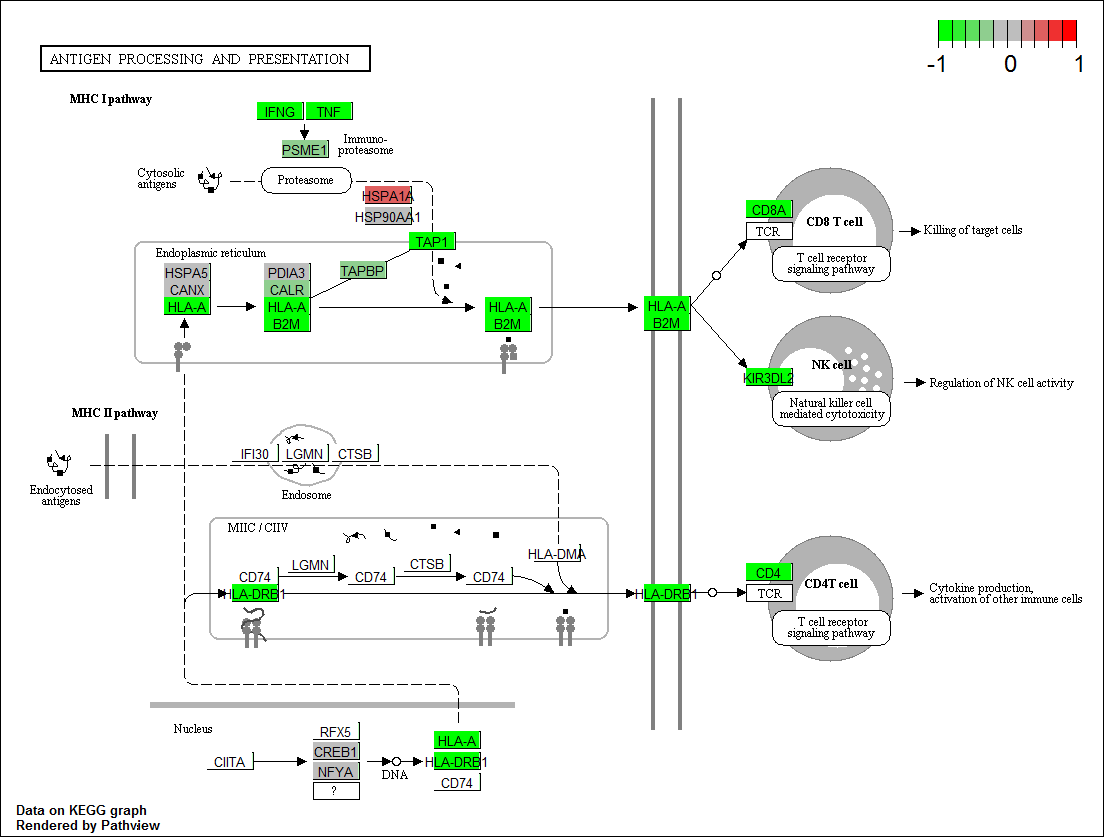

Supplement: S5 File — (ZIP) [file pone.0207799.s005.zip › S5_File/hsa04612.Tum_Tum4.png]

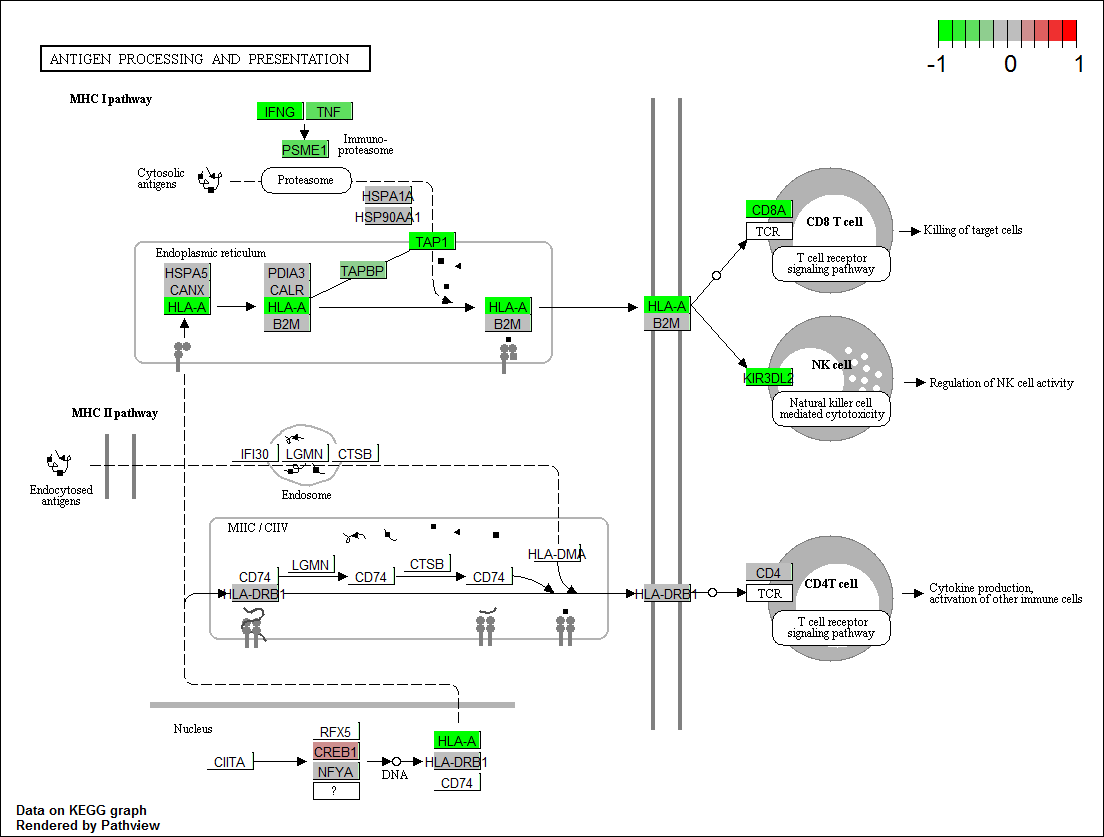

Supplement: S5 File — (ZIP) [file pone.0207799.s005.zip › S5_File/hsa04612.Tum_Tum5.png]

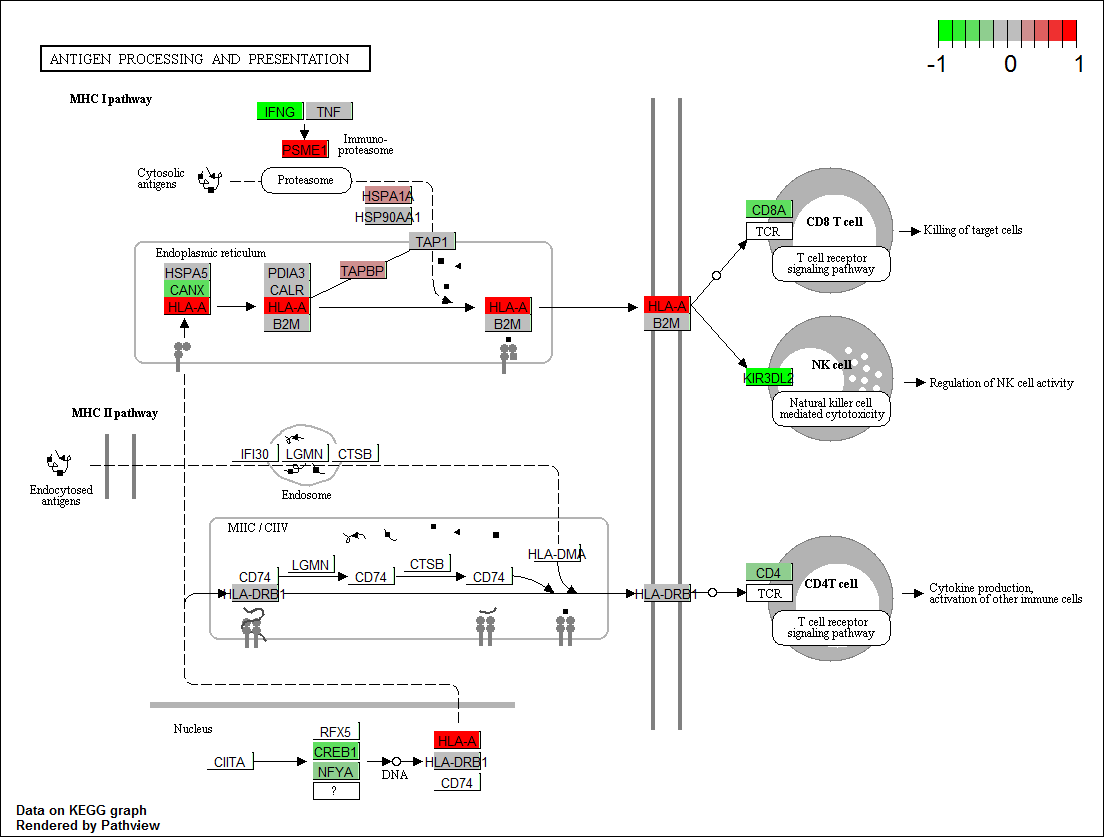

Supplement: S5 File — (ZIP) [file pone.0207799.s005.zip › S5_File/hsa04612.Tum_Tum6.png]

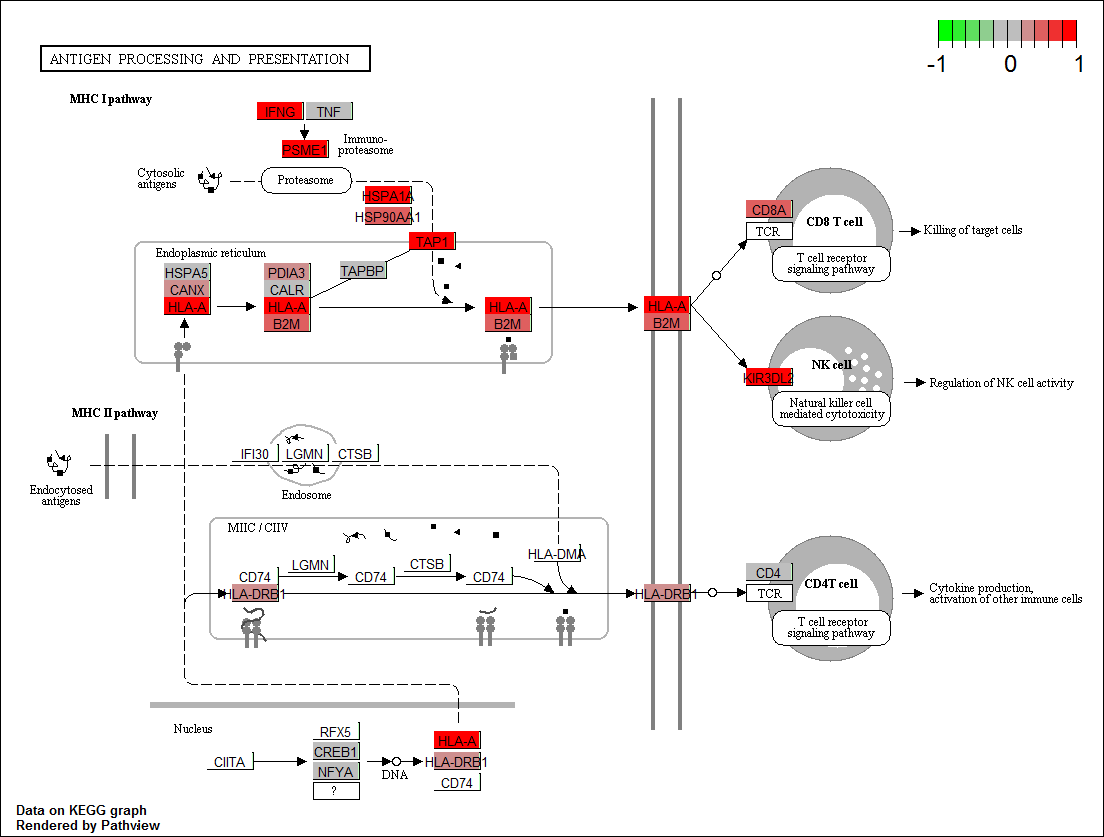

Supplement: S5 File — (ZIP) [file pone.0207799.s005.zip › S5_File/hsa04612.Tum_Tum7.png]

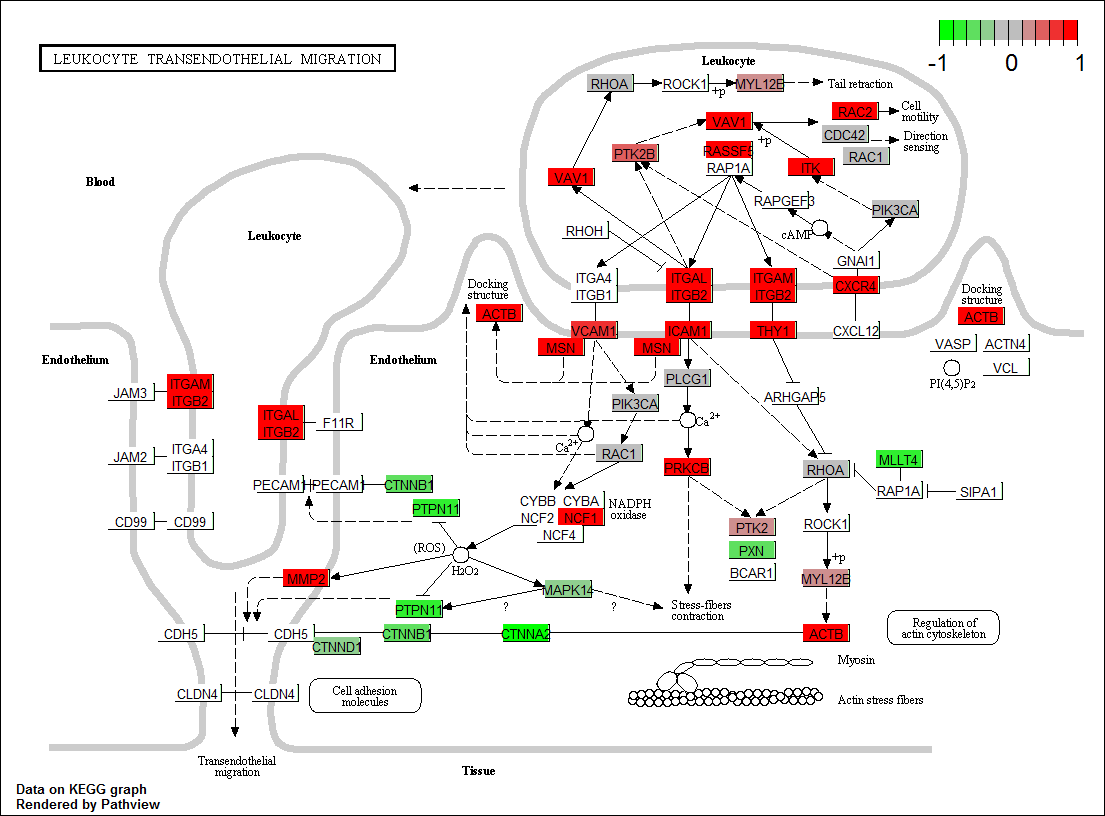

Supplement: S5 File — (ZIP) [file pone.0207799.s005.zip › S5_File/hsa04670.Tum_Nor1.png]

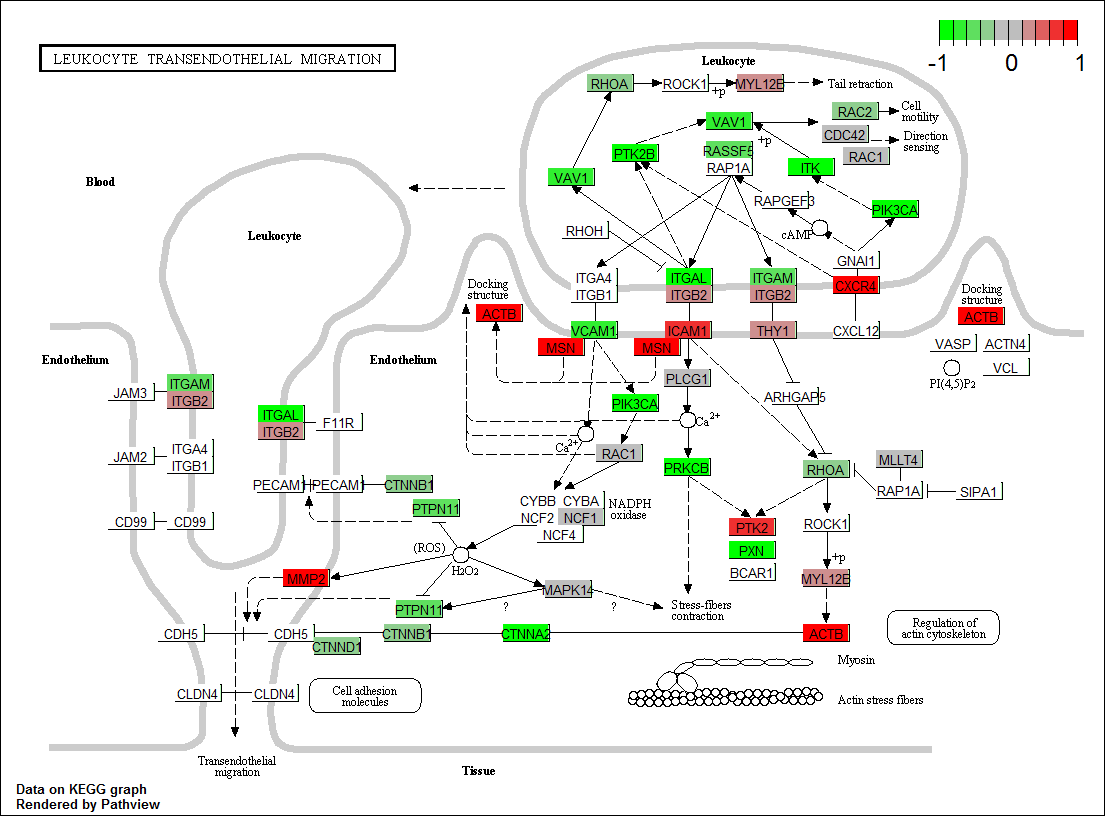

Supplement: S5 File — (ZIP) [file pone.0207799.s005.zip › S5_File/hsa04670.Tum_Nor2.png]

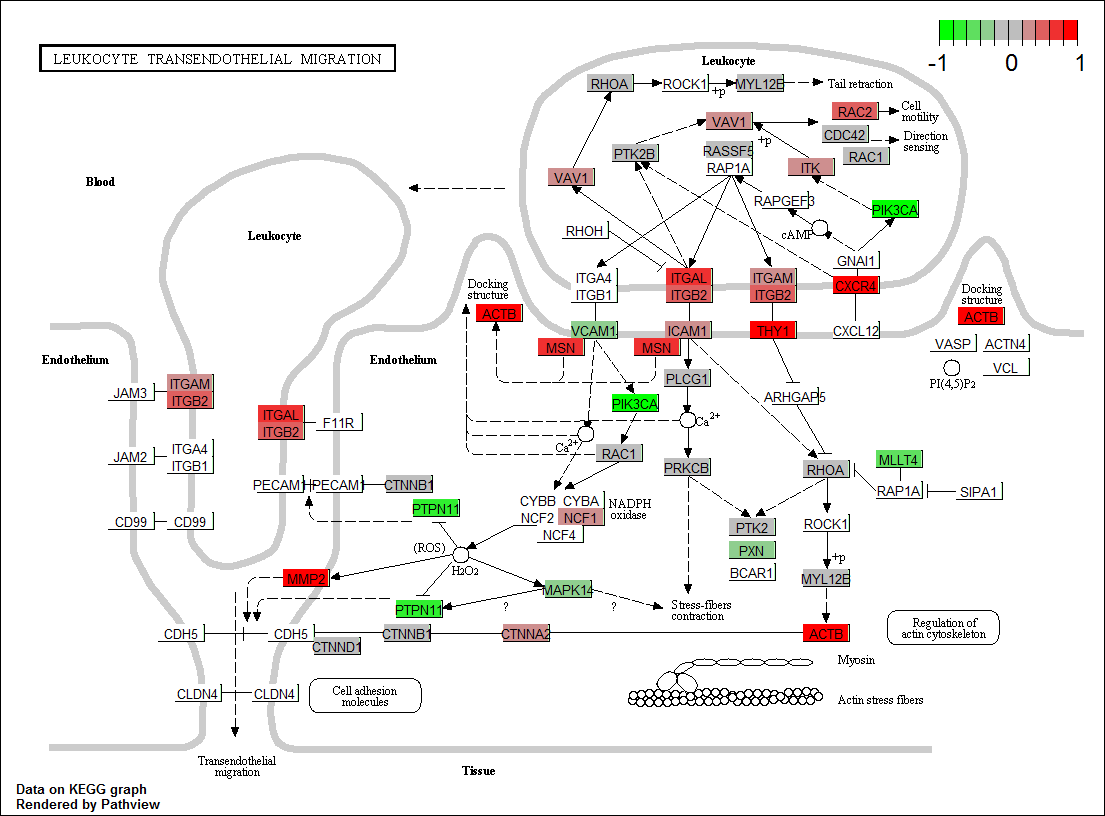

Supplement: S5 File — (ZIP) [file pone.0207799.s005.zip › S5_File/hsa04670.Tum_Nor3.png]

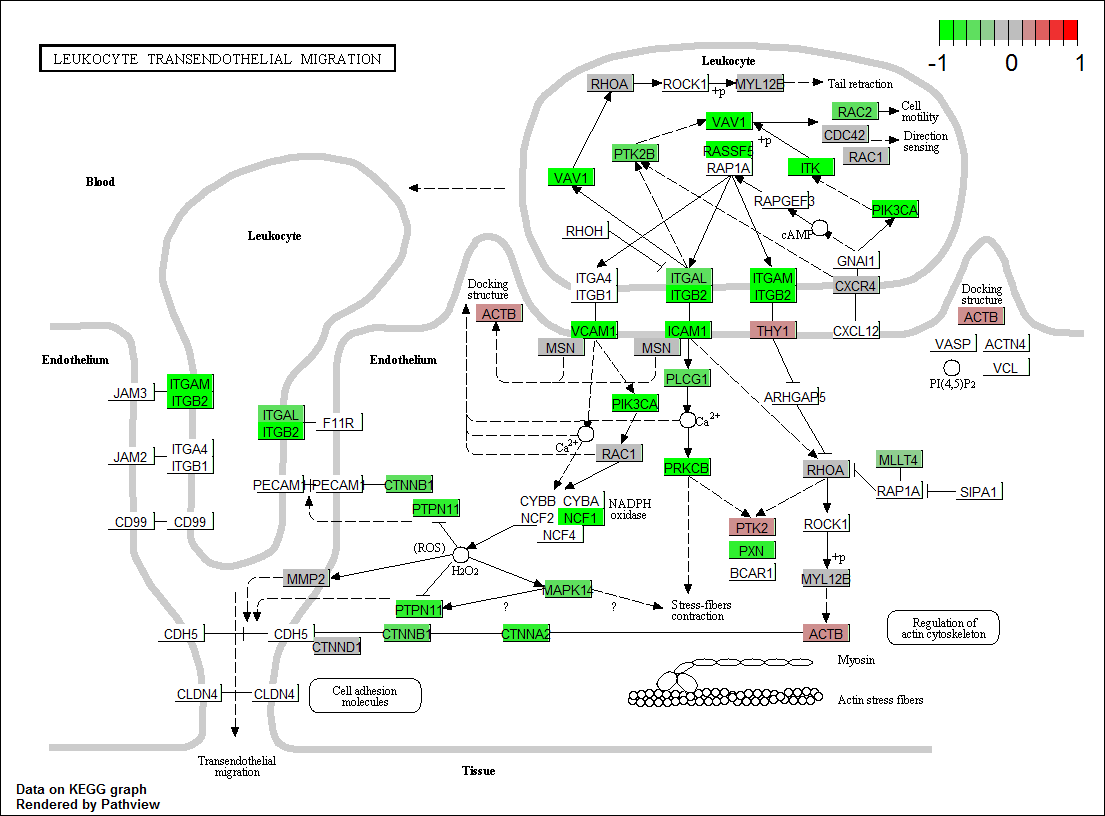

Supplement: S5 File — (ZIP) [file pone.0207799.s005.zip › S5_File/hsa04670.Tum_Nor4.png]

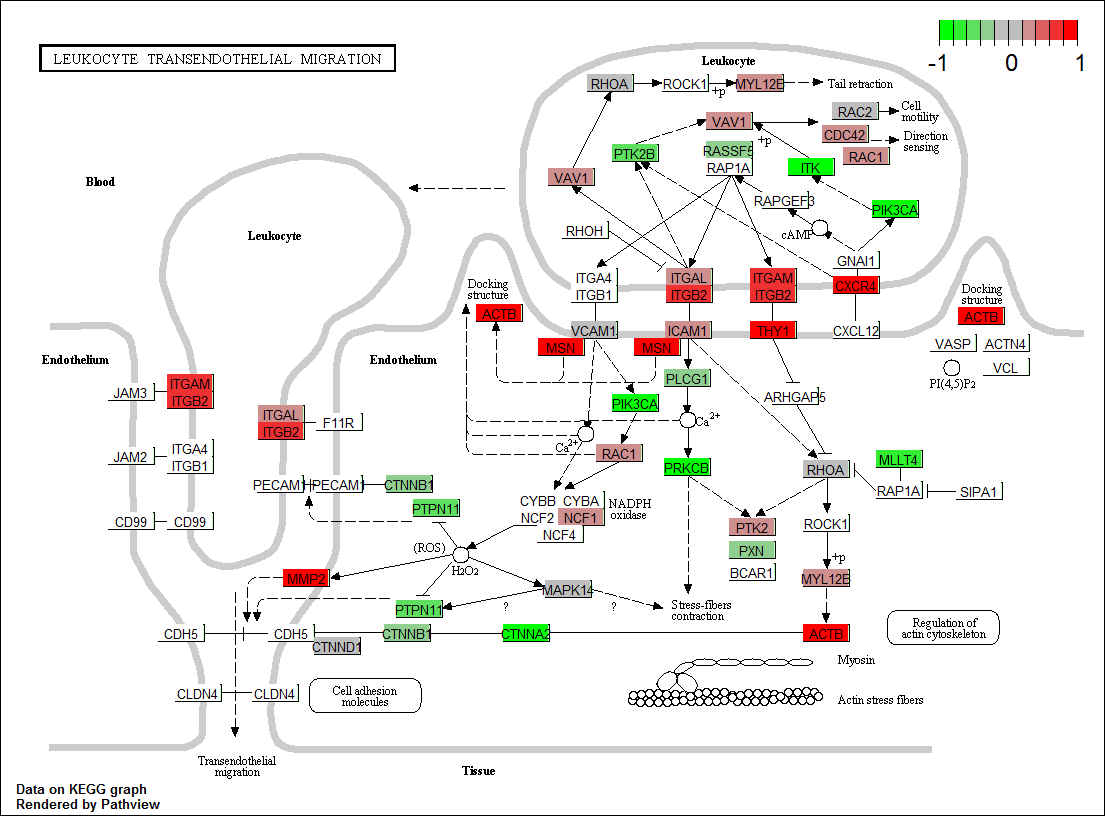

Supplement: S5 File — (ZIP) [file pone.0207799.s005.zip › S5_File/hsa04670.Tum_Nor5.png]

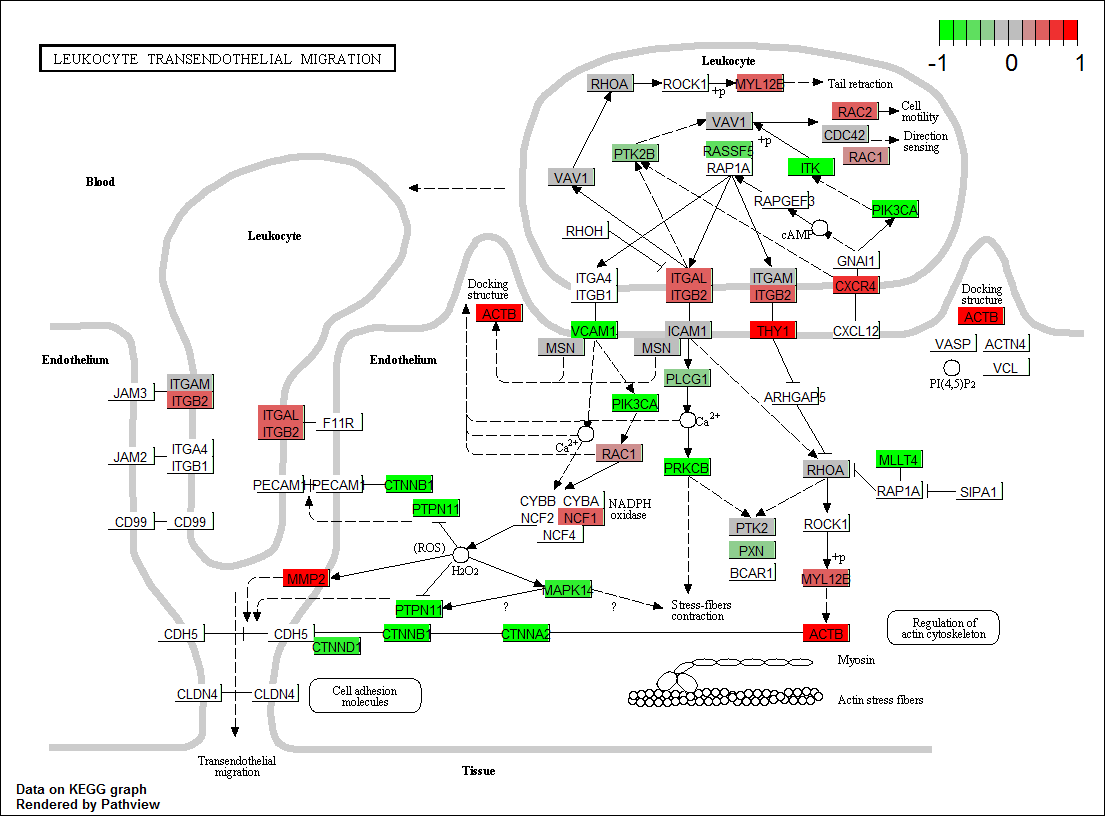

Supplement: S5 File — (ZIP) [file pone.0207799.s005.zip › S5_File/hsa04670.Tum_Nor6.png]

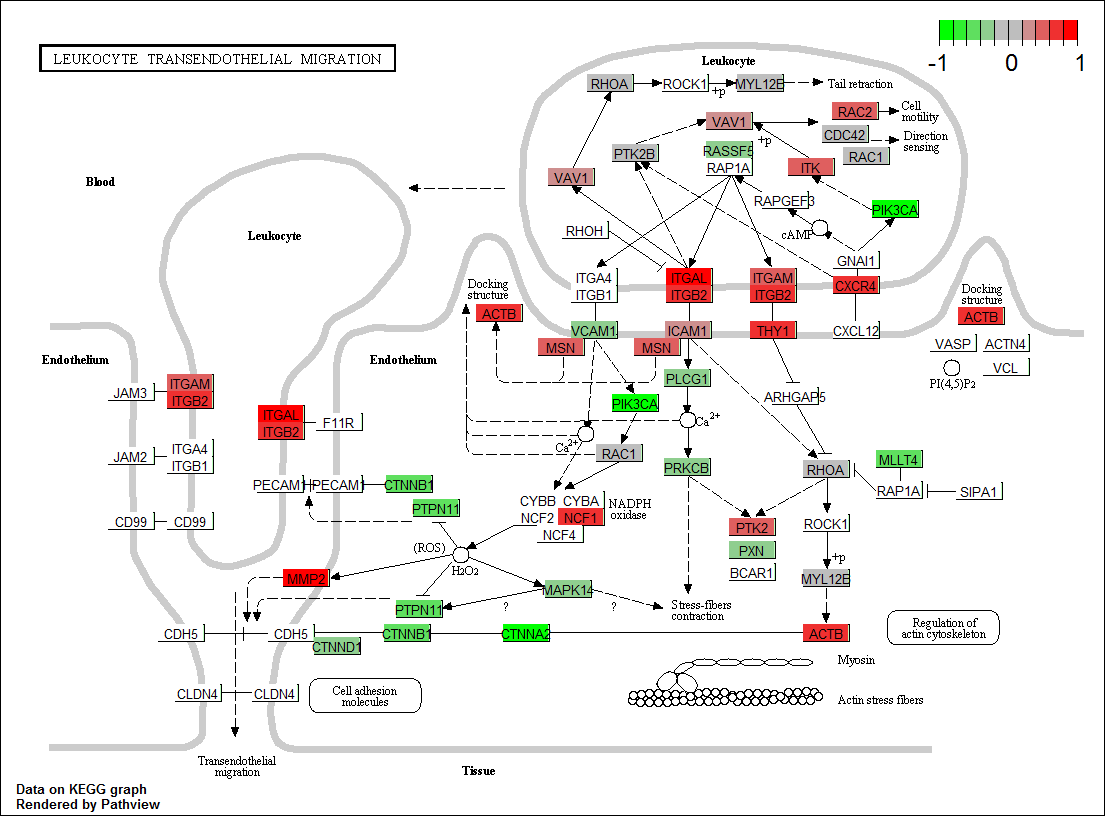

Supplement: S5 File — (ZIP) [file pone.0207799.s005.zip › S5_File/hsa04670.Tum_Nor7.png]

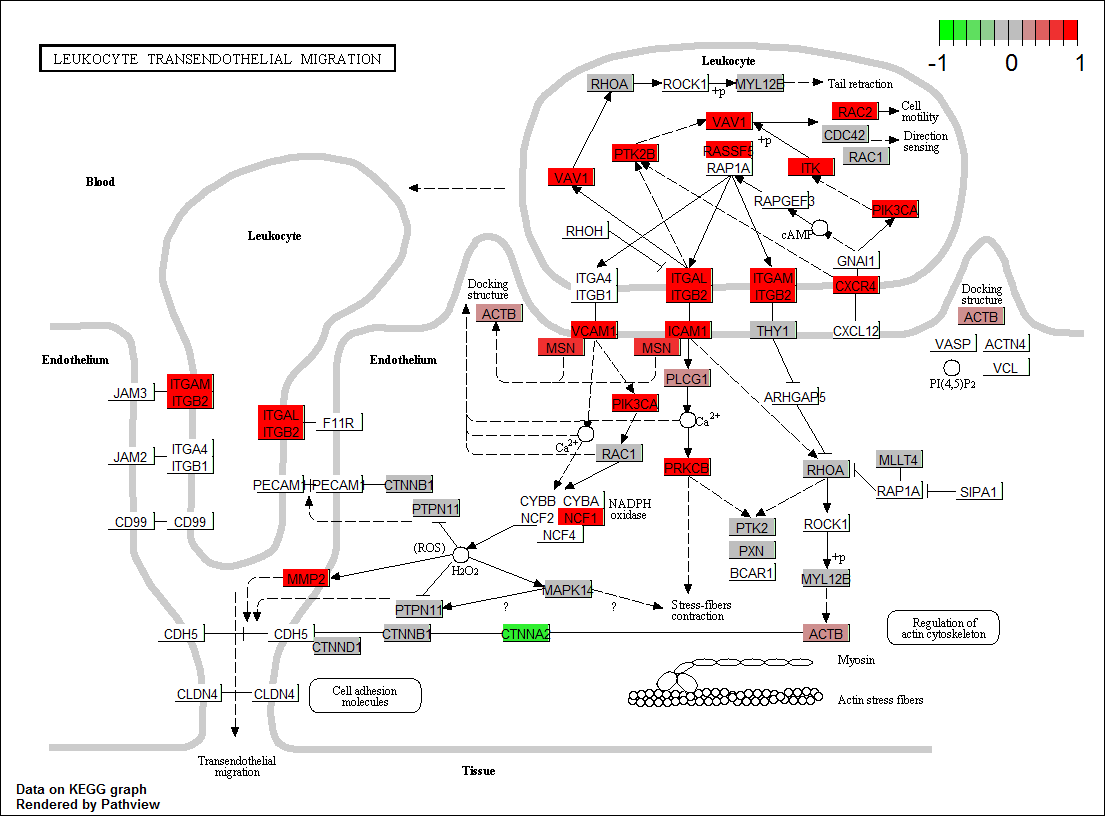

Supplement: S5 File — (ZIP) [file pone.0207799.s005.zip › S5_File/hsa04670.Tum_Tum1.png]

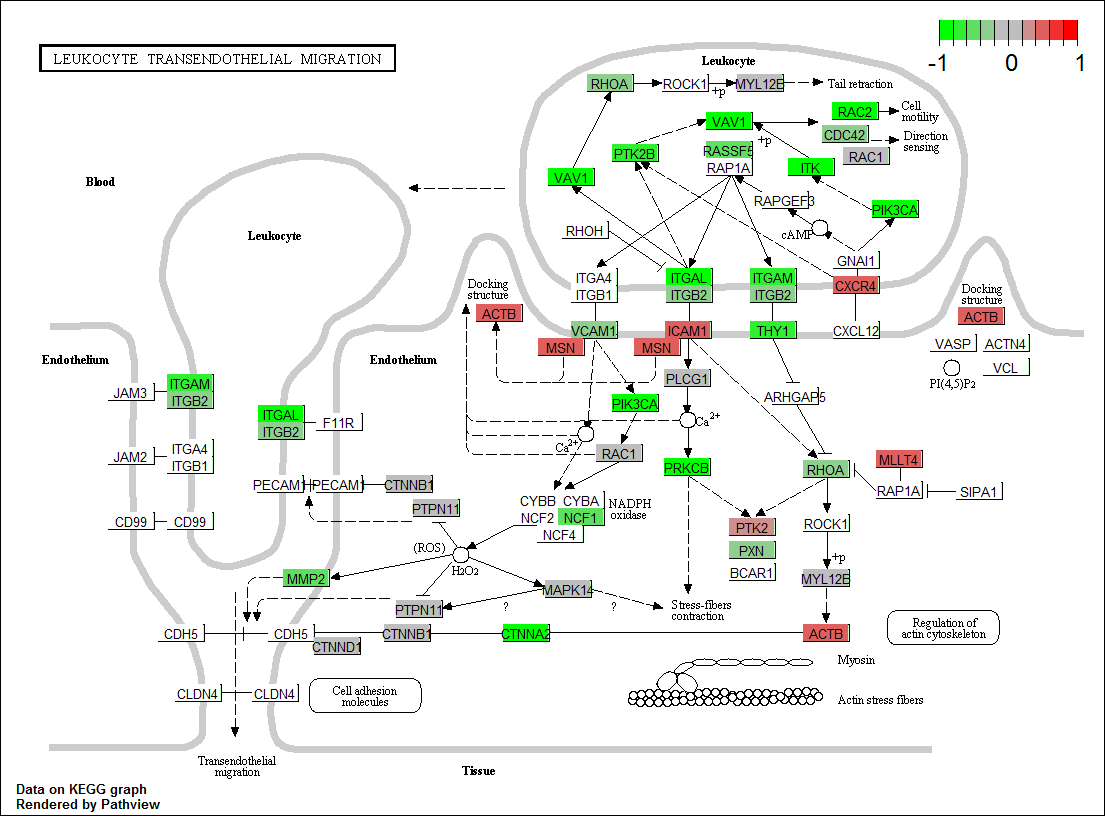

Supplement: S5 File — (ZIP) [file pone.0207799.s005.zip › S5_File/hsa04670.Tum_Tum2.png]

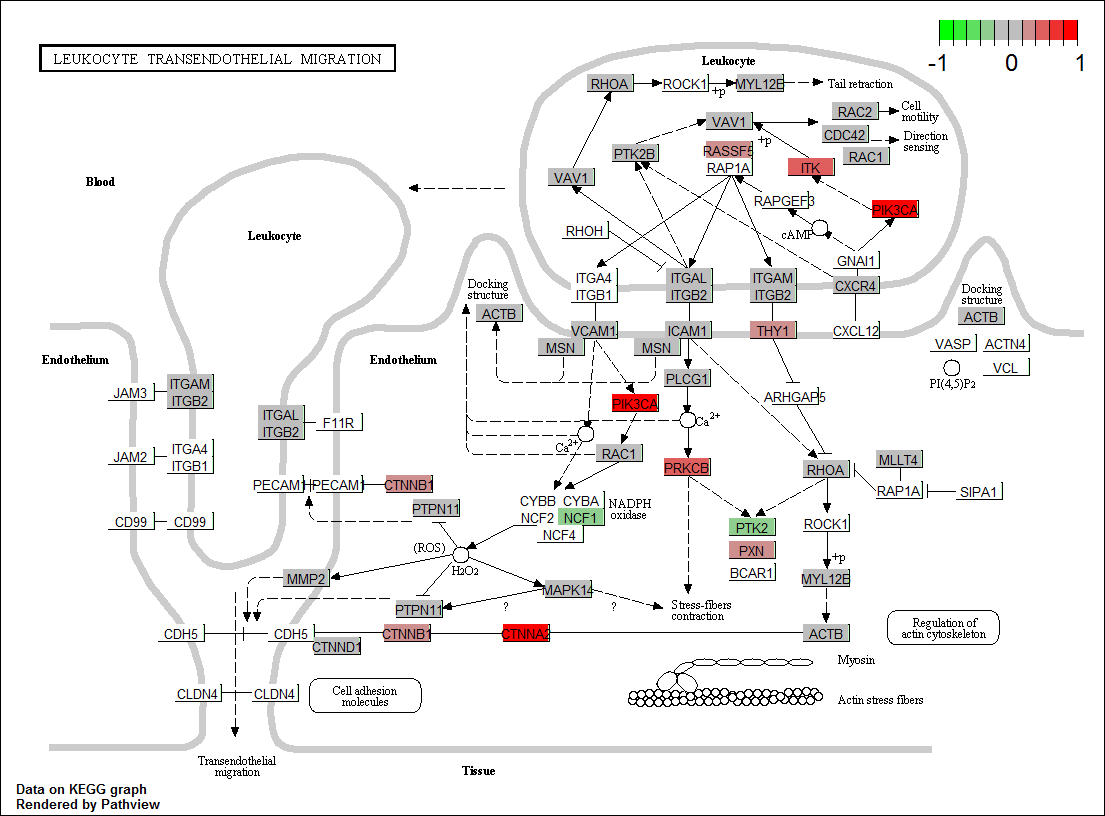

Supplement: S5 File — (ZIP) [file pone.0207799.s005.zip › S5_File/hsa04670.Tum_Tum3.png]

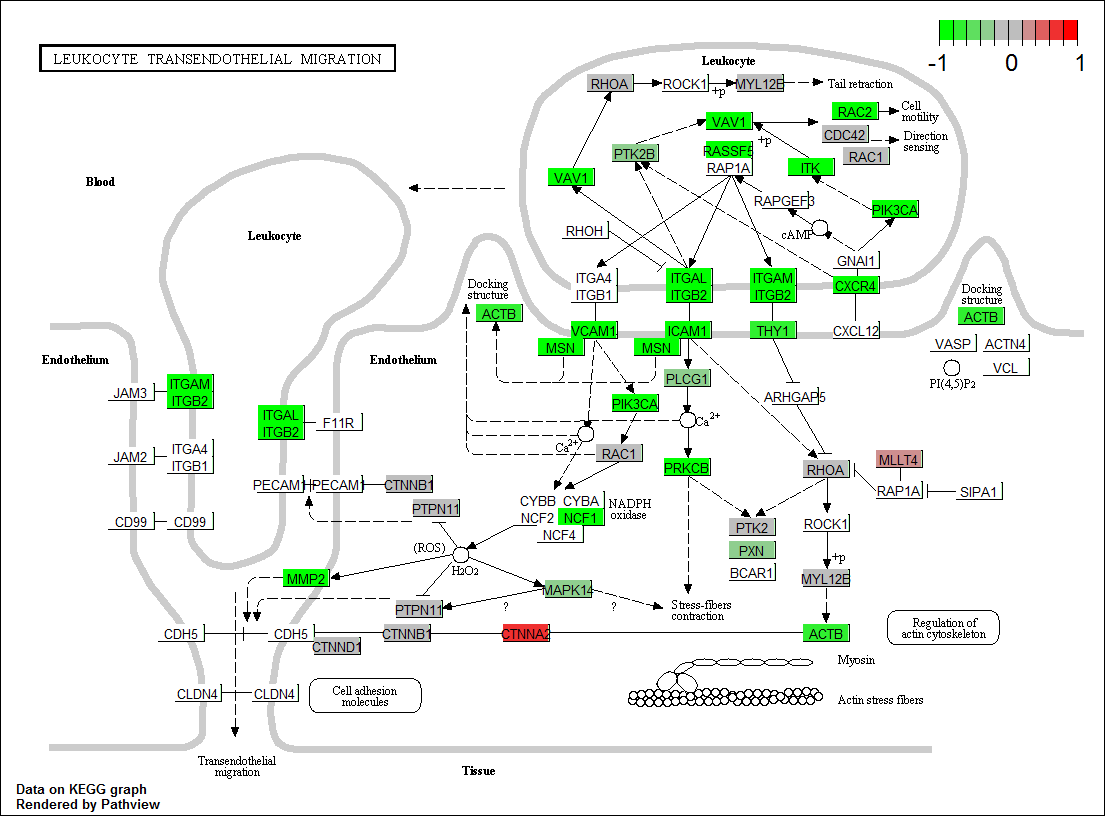

Supplement: S5 File — (ZIP) [file pone.0207799.s005.zip › S5_File/hsa04670.Tum_Tum4.png]

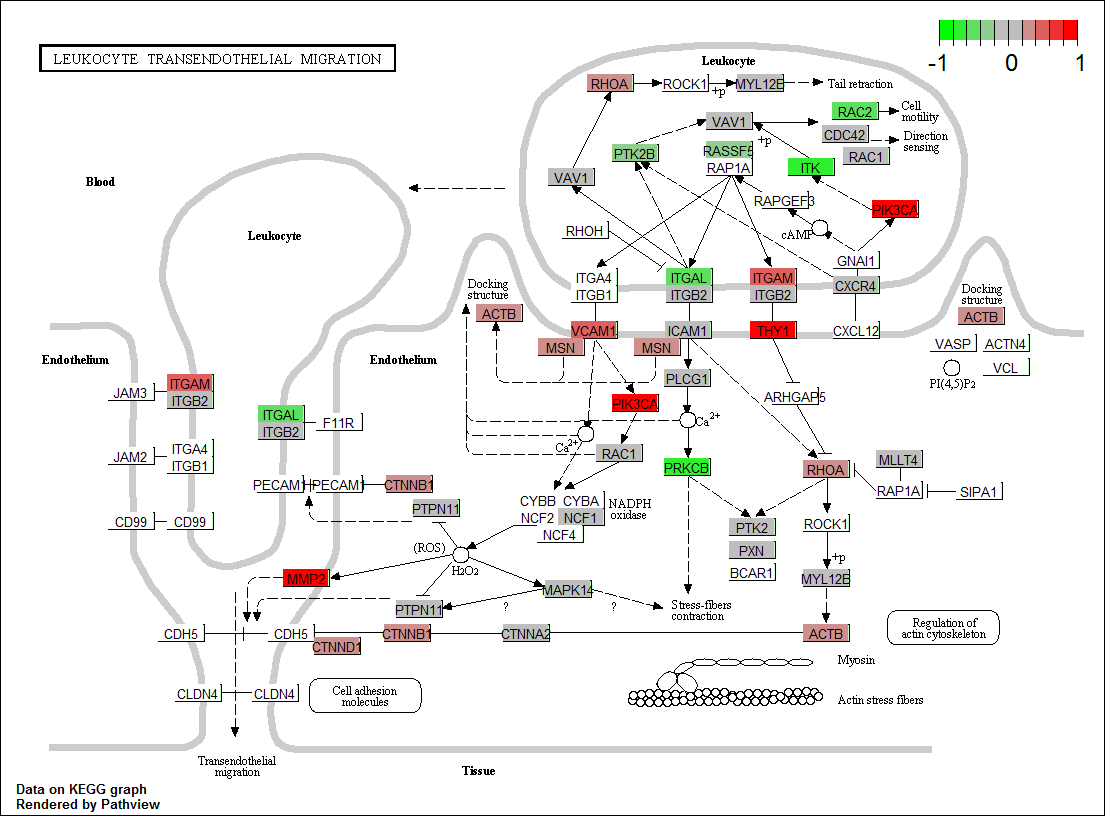

Supplement: S5 File — (ZIP) [file pone.0207799.s005.zip › S5_File/hsa04670.Tum_Tum5.png]

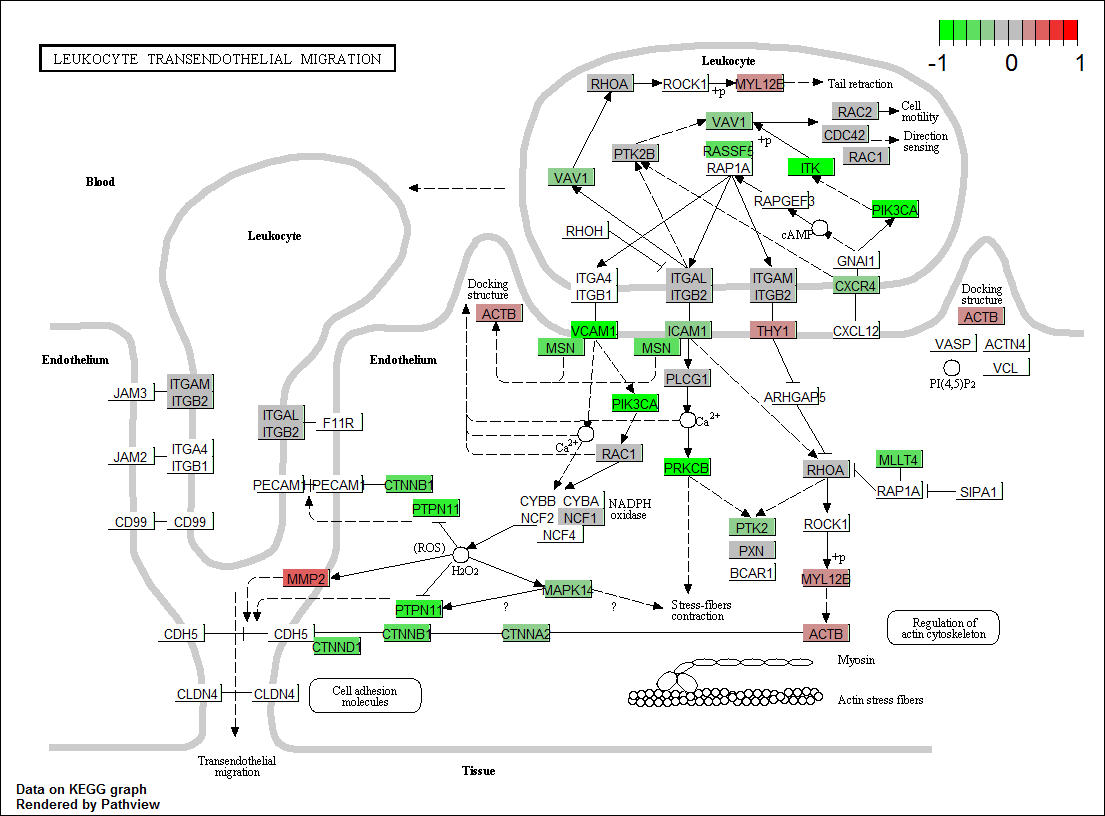

Supplement: S5 File — (ZIP) [file pone.0207799.s005.zip › S5_File/hsa04670.Tum_Tum6.png]

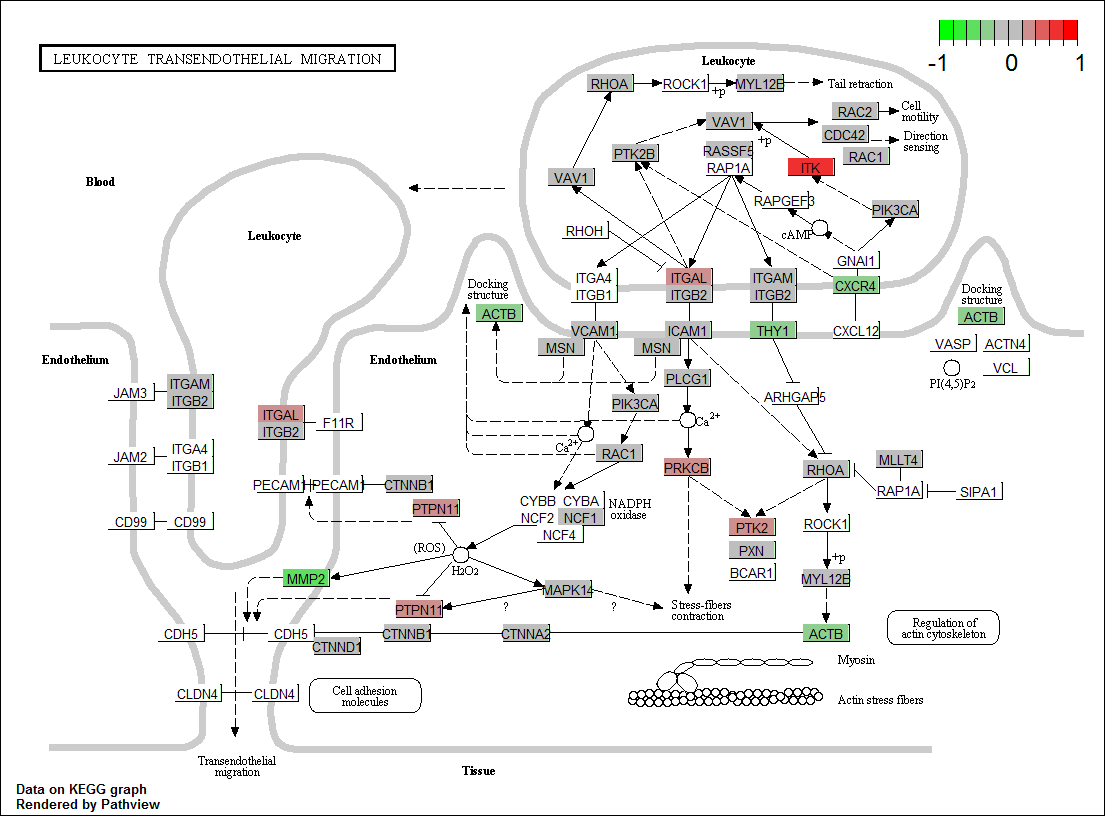

Supplement: S5 File — (ZIP) [file pone.0207799.s005.zip › S5_File/hsa04670.Tum_Tum7.png]
