# Supplementary material for: Subgrouping breast cancer patients based on immune evasion mechanisms unravels a high involvement of transforming growth factor-beta and decoy receptor 3
Source: PLoS One. 2018 Dec 4;13(12):e0207799. doi: 10.1371/journal.pone.0207799 (PMC6279052; doi:10.1371/journal.pone.0207799)
Supplement: S7 File — (DOCX) [file pone.0207799.s007.docx]

Patients in cluster 1 showed a high expression of molecules involved in antigen processing and presentation, leukocyte recruitment, and activation of immune cells compared to normal (Fig. 2A; Fig. 2B; Supplementary file 6, Cluster 1). However, several immune-inhibitory molecules were up-regulated. Molecules involved in immune tolerance (M2) such as CTLA4, PD-L1 and PD-1, and IFN-γ; and immune suppression (M1) such as IL-10, TGF-β1, and the Treg recruiting chemokine CCL22, were all significantly higher than normal. Tregs were recruited rather than induced in this subtype as the co-stimulatory tumor necrosis factor receptor TNFRSF9, which prevents the induction of naïve T-helper cells to Tregs (iTreg) by tumor microenvironment [1,2], was significantly up-regulated. Interferon gamma (IFN-γ) was among the highly-expressed genes in cluster 1 patients. Moreover, resistance to apoptosis (M3) manifested by the upregulated expression of anti-apoptotic molecules BIRC3, TRAF1, TNFAIP3, and the counterattack (M4) by DcR3, were unmissable.

The expression of antigen presenting molecules in cluster 2 was mostly up-regulated. HLA-A was up-regulated but both HLA-B and beta-2-microglobulin (B2M) were not differentially expressed from normal (Supplementary file 6). To check if antigen presentation was impaired we examined the downstream steps of the cancer-immunity cycle. The expression of adhesion molecules was weak as almost all molecules were either not differentially expressed (ITGB2, ITGB7, THY1, and SELL) or significantly lower than the normal mean expression (ITGAL, VCAM, SELPLG, and SELP) (Supplementary file 6, Cluster 2). The non-upregulated expression indicates that adhesion molecules were not stimulated, explaining the low recruitment of T-helper and CTL as their markers CD4 and CD8A were not up-regulated (Supplementary file 6). Overall, this indicates that antigen presentation was impaired (M5).

The expression of antigen presenting molecules in cluster 3 was mostly not higher than normal. Specifically, B2M, HLA-A, and HLA-B were not differentially expressed (Supplementary file 6, Cluster 3). The low expression of MHC-I did not increase NK cell recruitment as the corresponding markers, KIR3DL2 and KLRD1, were not increased. The weak antigen presentation caused an inefficient recruitment of T-helper cells and CTLs (CD4 and CD8A) and deficient activation of CTL as a single cytotoxic molecule, GZMA, was up-regulated. TGF-β1 and DcR3 were up-regulated in this subtype. Thus, impaired antigen presentation (M5), immunosuppression (M1) and counterattack were the mechanisms of evasion in this subtype. The up-regulated expression of TGF-β1 (M1) decreases the expression of MHC-I and II molecules, inhibits the expression of B2M and HLA-DR, and results in decreased antigen presentation [3–8]. TGF-β also inhibits CTL activation, proliferation, and differentiation [9,10]; and the transcription of CTL’s cytotoxic molecules, TCR components: ZAP70 and ITK [11,12]. In addition, DcR3 (M4) increases invasion and migration of BRCA tumors, inhibits T cell activation and chemotaxis [13,14], and suppresses the activation and differentiation of DCs and macrophages, and alters the latter’s phagocytic activity [15,16].

TNFRSF9, CCL22, and CCR4 were up-regulated but CTLA4 was not, thus, Tregs were not present. TNFRSF9 however, induces T cell apoptosis after activation (anergy) which is another mechanism to decrease CTL availability [17,18].

None of the cancer-immunity cycle steps were activated in cluster 4 (Supplementary file 6, Cluster 4). Thus, evasion in cluster 4 was caused by the lack of a danger signal, *aka* ignorance (M6).

The expression of molecules involved in the antigen presentation machinery was mostly not higher than normal in cluster 5. Both T cell markers and CTL cytotoxic molecules were not up-regulated but TGF-β1 was (Supplementary file 6, Cluster 5). Thus, evasion in cluster 5 was potentially caused by TGF-β1-induced impairment of antigen presentation (M1 and M5). The up-regulated expression of FOXP3 but not CTLA4 can be explained by increased expression of TGF-β1 and TNFRSF9. TGF-β inhibits CTL’s activity by transforming naïve T helper cells to regulatory T cells (Tregs) by increasing naïve T helper expression of FOXP3 [19]. However, since TNFRSF9 prevents Treg induction by the tumor microenvironment, these T cells cannot prohibit the CTLA4 marker.

B2M in cluster 6 was not differentially expressed. Some leukocyte recruiting molecules were higher than normal (ITGAL, ITGB2 ITGB7, and THY1), whereas the rest were not upregulated, and all immune cell markers and cytotoxic molecules were not upregulated compared to normal (Supplementary file 6, Cluster 6). This indicates that there was an impaired antigen presentation (M5) that resulted in impairment in leukocyte recruitment and subsequent activation.

CTLA4, TGF-β1, and DcR3 were higher than the normal mean expression indicating tolerance (M2), immunosuppression (M1), and counterattack (M4).

In cluster 7 most of the genes involved in antigen presentation had a higher mean expression than normal. The expression of the majority of adhesion molecules was significantly higher than normal. CD4^+^ T-helper cells were not recruited to the tumor microenvironment whereas CD8^+^ CTLs were, as their marker (CD8A) and cytotoxic molecules were up-regulated.

It seems that Tregs were recruited to the tumor microenvironment as CTLA4, TNFRSF9, CCL22, and CCR4 were up-regulated (Supplementary file 6, Cluster 7). IFN-γ has contradictory functions with either an anti-tumor or a pro-tumor effect [20]. As an anti-tumor molecule, IFN-γ increases immune cell recruitment and causes a direct inhibition of tumor growth and recognition and elimination by the immune system [20,21]. Other studies on IFN-γ pointed to a pro-tumor role, wherein it was shown to increase Treg development, decrease neutrophil infiltration, aide in tumor proliferation and resistance to apoptosis by CTL and NK cells, and increase PD-L1 expression [20,22]. In both clusters 1 and 7, Treg development was increased (CTLA4) and PD-L1 and anti-apoptotic molecules were up-regulated in cluster 1, showing a pro-tumor effect potentially caused by IFN-γ.

**List of abbreviations:**

B2M: Beta-2 microglobulin

BRCA: Breast Cancer

BIRC3: Baculoviral IAP repeat-containing protein 3

CCL22: C-C motif chemokine 22

CCR4: C-C chemokine receptor type 4

CD4: Cluster of differentiation 4

CD8: Cluster of differentiation 8

CTL: Cytotoxic T lymphocyte

CTLA4: Cytotoxic T lymphocyte-associated protein 4

DC: Dendritic cells

DcR3: Decoy receptor 3

FOXP3: Forkhead box P3

GZMA: Granzyme A

HLA-A: Major histocompatibility complex, class I, A

HLA-B: Major histocompatibility complex, class I, B

IFN-γ: Interferon gamma

IL-10: Interleukin 10

ITGB: Integrin beta

ITK: Interleukin-2-inducible T-cell kinase

KIR3DL2: Killer cell immunoglobulin-like receptor 3DL2

KLRD1: Killer cell lectin-like receptor subfamily D, member 1

MHC-I: Major histocompatibility complex, class I

NK: Natural killer

PD-1: Programmed cell death protein 1

PD-L1: Programmed cell death protein 1 ligand

SELP: P-selectin

SELPLG: Selectin P ligand

TCR: T-cell receptor

TGF-β: Transforming growth factor beta

TGF-β1: Transforming growth factor beta 1

TGF-β2: Transforming growth factor beta 2

THY1: Thymocyte antigen 1

TNFAIP3: Tumor necrosis factor, alpha-induced protein 3

TNFRSF9: Tumor necrosis factor receptor superfamily member 9

TRAF1: TNF receptor-associated factor 1

VCAM: Vascular cell adhesion molecule

ZAP70: Zeta-chain-associated protein kinase 70

**References:**

1. Bremer E. Targeting of the tumor necrosis factor receptor superfamily for cancer immunotherapy. ISRN Oncol 2013;2013:371854. doi:10.1155/2013/371854.

2. Gooden MJM, de Bock GH, Leffers N, Daemen T, Nijman HW. The prognostic influence of tumour-infiltrating lymphocytes in cancer: a systematic review with meta-analysis. Br J Cancer 2011;105:93–103. doi:10.1038/bjc.2011.189.

3. Geiser AG, Letterio JJ, Kulkarni AB, Karlsson S, Roberts AB, Sporn MB. Transforming growth factor beta 1 (TGF-beta 1) controls expression of major histocompatibility genes in the postnatal mouse: aberrant histocompatibility antigen expression in the pathogenesis of the TGF-beta 1 null mouse phenotype. Proc Natl Acad Sci U S A 1993;90:9944–8.

4. Ma D, Niederkorn JY. Transforming growth factor-beta down-regulates major histocompatibility complex class I antigen expression and increases the susceptibility of uveal melanoma cells to natural killer cell-mediated cytolysis. Immunology 1995;86:263–9.

5. Johns LD, Babcock G, Green D, Freedman M, Sriram S, Ransohoff RM. Transforming growth factor-??1 differentially regulates proliferation and MHC class-II antigen expression in forebrain and brainstem astrocyte primary cultures. Brain Res 1992;585:229–36. doi:10.1016/0006-8993(92)91211-V.

6. Lee YJ, Han Y, Lu HT, Nguyen V, Qin H, Howe PH, et al. TGF-beta suppresses IFN-gamma induction of class II MHC gene expression by inhibiting class II transactivator messenger RNA expression. J Immunol 1997;158:2065–75.

7. Bierie B, Moses HL. Tumour microenvironment: TGFbeta: the molecular Jekyll and Hyde of cancer. Nat Rev Cancer 2006;6:506–20. doi:10.1038/nrc1926.

8. Li Y, Han B, Li K, Jiao LR, Habib N, Wang H. TGF-β 1 inhibits HLA-DR and β 2-microglobulin expression in HeLa cells induced with r-IFN. Transplant. Proc., vol. 31, Elsevier; 1999, p. 2143–5.

9. Ranges GE, Figari IS, Espevik T, Palladino MA. Inhibition of cytotoxic T cell development by transforming growth factor beta and reversal by recombinant tumor necrosis factor alpha. J Exp Med 1987;166:991–8.

10. Kehrl JH, Wakefield LM, Roberts a B, Jakowlew S, Alvarez-Mon M, Derynck R, et al. Production of transforming growth factor beta by human T lymphocytes and its potential role in the regulation of T cell growth. J Exp Med 1986;163:1037–50. doi:10.1084/jem.163.5.1037.

11. Töpfer K, Kempe S, Müller N, Schmitz M, Bachmann M, Cartellieri M, et al. Tumor evasion from T cell surveillance. J Biomed Biotechnol 2011;2011:918471. doi:10.1155/2011/918471.

12. Di Bari MG, Lutsiak MEC, Takai S, Mostbock S, Farsaci B, Tolouei Semnani R, et al. TGF-β modulates the functionality of tumor-infiltrating CD8 + T cells through effects on TCR signaling and Spred1 expression. Cancer Immunol Immunother 2009;58:1809–18. doi:10.1007/s00262-009-0692-9.

13. Shi G, Wu Y, Zhang J, Wu J. Death decoy receptor TR6/DcR3 inhibits T cell chemotaxis in vitro and in vivo. J Immunol 2003;171:3407–14. doi:10.4049/jimmunol.171.7.3407.

14. ZHICHENG GE1, 2, ANDREW J. SANDERS1, LIN YE1 REM and WGJ, 1Metastasis. Expression of death receptor-3 in human breast cancer and its functional effects on breast cancer cells in vitro. Oncol Rep 2013;29:1356–64. doi:10.3892/or.2013.2259.

15. Hsu T-L, Chang Y-C, Chen S-J, Liu Y-J, Chiu AW, Chio C-C, et al. Modulation of dendritic cell differentiation and maturation by decoy receptor 3. J Immunol 2002;168:4846–53. doi:10.4049/jimmunol.168.10.4846.

16. Chang Y-C, Hsu T-L, Lin H-H, Chio C-C, Chiu AW, Chen N-J, et al. Modulation of macrophage differentiation and activation by decoy receptor 3. J Leukoc Biol 2004;75:486–94. doi:10.1189/jlb.0903448.

17. Schwarz H, Blanco FJ, von Kempis J, Valbracht J, Lotz M. ILA, a member of the human nerve growth factor/tumor necrosis factor receptor family, regulates T-lymphocyte proliferation and survival. Blood 1996;87:2839–45.

18. Michel J, Pauly S, Langstein J, Krammer PH, Schwarz H. CD137-induced apoptosis is independent of CD95. Immunology 1999;98:42–6. doi:10.1046/j.1365-2567.1999.00851.x.

19. Chen W, Jin W, Hardegen N, Lei K-J, Li L, Marinos N, et al. Conversion of peripheral CD4+CD25- naive T cells to CD4+CD25+ regulatory T cells by TGF-beta induction of transcription factor Foxp3. J Exp Med 2003;198:1875–86. doi:10.1084/jem.20030152.

20. Zaidi MR, Merlino G. The two faces of interferon-γ in cancer. Clin Cancer Res 2011;17:6118–24. doi:10.1158/1078-0432.CCR-11-0482.

21. Patel SJ, Sanjana NE, Kishton RJ, Eidizadeh A, Vodnala SK, Cam M, et al. Identification of essential genes for cancer immunotherapy. Nature 2017;000. doi:10.1038/nature23477 [doi].

22. Rozali EN, Hato S V., Robinson BW, Lake RA, Lesterhuis WJ. Programmed death ligand 2 in cancer-induced immune suppression. Clin Dev Immunol 2012;2012. doi:10.1155/2012/656340.
