# Supplementary material for: Subgrouping breast cancer patients based on immune evasion mechanisms unravels a high involvement of transforming growth factor-beta and decoy receptor 3
Source: PLoS One. 2018 Dec 4;13(12):e0207799. doi: 10.1371/journal.pone.0207799 (PMC6279052; doi:10.1371/journal.pone.0207799)
Supplement: S9 File — (DOCX) [file pone.0207799.s009.docx]

**Classification with Random Forest**

In the main text, a classification tree was fit in order to find important biomarker genes to distinguish the 7 clusters and assist the study of different immune evasion mechanisms. We also mentioned that using a classification tree is due to its intuitive output with straightforward clinical interpretations. All biomarker genes with their cutoff values are displayed in Figure 3. As a matter of fact, we need to point out that many machine learning algorithms can be used for the purpose of clustering and classification. A classification tree may not be optimal in terms of prediction accuracy and statistical performance. To accommodate for this limitation, we also tried a random forest model.

A random forest, also known as a random decision forest [1,2], is a collection of multiple decision trees. It employs the idea of Bootstrap resampling [3,4], with majority voting (for classification) and mean prediction (for regression). A random forest can overcome the usual problem of overfitting in a decision tree. Further explanation can be found in [5].

We used the R package *randomForest*. Candidate biomarker genes are defined as the top features selected by a mean decrease in accuracy and Gini coefficient in the model. Table S9-1 displays the complete confusion matrix and Table S9-2 gives the per-cluster sensitivity and specificity. The model performance seems to be stably satisfactory. The overall accuracy of this model is 0.847 with a 95% confidence interval (0.824, 0.868). To visually investigate the quality of this model fitting, we also created summary plots from the model. Figure S9-1 is a histogram of the frequency of all genes being used in the model. With a forest with 1,000 trees, the right-skewed shape of the histogram indicates that a small proportion of genes are used much more than the other genes. They may serve as potential biomarkers. A more quantitative way to detect biomarker genes is given by Figure S9-2. Top 20 most important genes in terms of mean decrease in accuracy and mean decrease in Gini index are plotted. We can also see that there is a substantial overlapping between the two lists. Compared with the biomarker selection performance by a single tree, we also find that they highly agree. In **Figure 3**, IL2RG, ABCB1, DCN, LCK, and SELP are nodes on a higher level close to the root, and they all show up to be important in the forest.

**Table S9-1**. Confusion Matrix of a random forest model fitting to the TCGA BRCA data. Rows in the table correspond to the true cluster labels and columns correspond to predicted labels.

|  | Cluster1 | Cluster2 | Cluster3 | Cluster4 | Cluster5 | Cluster6 | Cluster7 | Other |
| --- | --- | --- | --- | --- | --- | --- | --- | --- |
| Cluster1 | 288 | 3 | 5 | 0 | 0 | 0 | 0 | 0 |
| Cluster2 | 3 | 84 | 0 | 0 | 0 | 0 | 0 | 0 |
| Cluster3 | 8 | 0 | 121 | 0 | 2 | 2 | 0 | 10 |
| Cluster4 | 0 | 0 | 0 | 91 | 0 | 2 | 0 | 15 |
| Cluster5 | 6 | 3 | 2 | 3 | 88 | 0 | 0 | 9 |
| Cluster6 | 3 | 1 | 3 | 1 | 1 | 28 | 6 | 17 |
| Cluster7 | 12 | 2 | 3 | 0 | 1 | 1 | 24 | 16 |
| Other | 0 | 3 | 8 | 7 | 5 | 0 | 0 | 178 |

**Table S9-2**. Per-cluster sensitivity and specificity of the random forest model.

|  | Cluster1 | Cluster2 | Cluster3 | Cluster4 | Cluster5 | Cluster6 | Cluster7 | Other |
| --- | --- | --- | --- | --- | --- | --- | --- | --- |
| Sensitivity | 0.9 | 0.875 | 0.852 | 0.892 | 0.907 | 0.848 | 0.8 | 0.726 |
| Specificity | 0.989 | 0.997 | 0.976 | 0.982 | 0.976 | 0.969 | 0.966 | 0.972 |


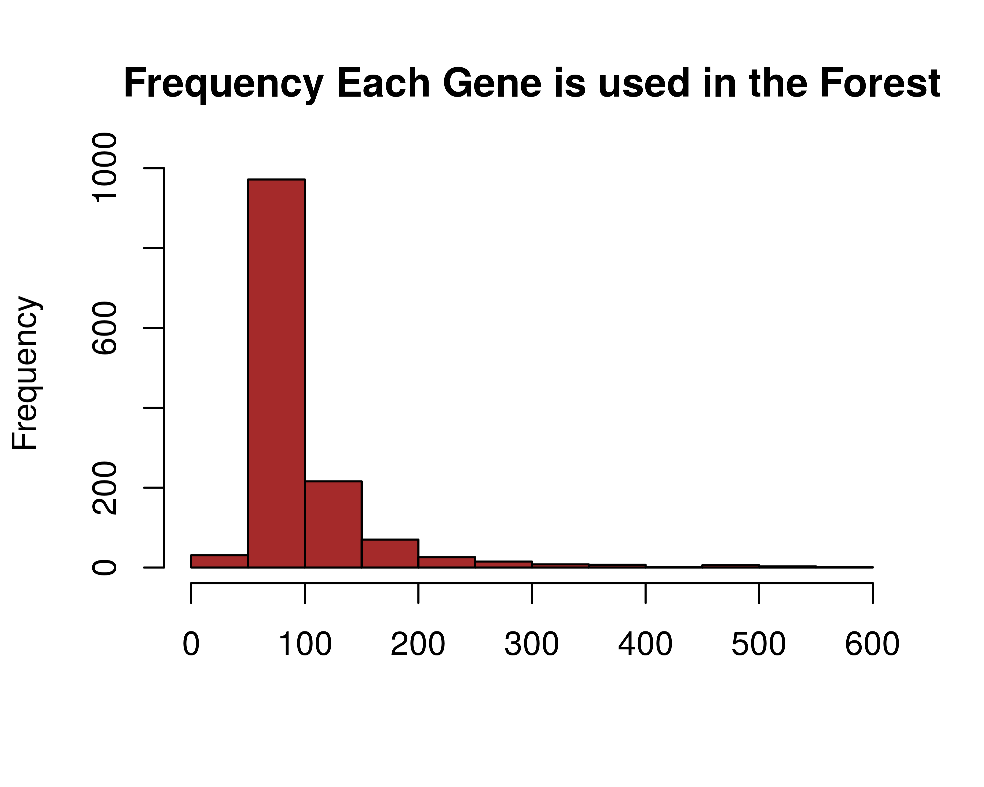


Figure S9-1. The frequency of each gene used in the forest





Figure S9-2. Feature/gene importance plot

As for concluding remarks, we want to point out that biclustering is an unsupervised learning algorithm, so after we identify subsets of patients (clusters), we need to verify the cluster labels by applying other popular machine learning algorithms for classification. Options are not limited to a classification tree or a random forest. Each method has its specific way of model fitting and feature selection. Which method to choose is totally subject to clinical researchers, who should take into consideration the statistical performance, practical interpretation, and affordability of the computational burden.

**References**

1. Tin Kam Ho. Random decision forests. Proc 3rd Int Conf Doc Anal Recognit 1995;1:278–82. doi:10.1109/ICDAR.1995.598994.

2. Barandiaran I. The Random Subspace Method for Constructing Decision Forests. Trans PATTERN Anal Mach Intell 1998;20.

3. Efron B and TRJ. An Introduction to the Bootstrap. CRC Press; 1994.

4. Efron B. Second Thoughts on the Bootstrap. Stat Sci 2003;18:135–40.

5. Breiman L. Random Forests. Mach Learn 2001;45:5–32. doi:10.1023/A:1010933404324.
